# First comprehensive analysis of virulence-associated gene profiles and co-occurrence patterns of avian pathogenic Escherichia coli isolated from chickens in northern Vietnam

**DOI:** 10.14202/vetworld.2026.3864-3882

**Published:** 2026-09-07

**Authors:** Van Truong Le, Quang Lam Truong, Thi Huong Giang Tran, Thi My Le Huynh, Thi Ngoc Vu, Thi Trang Le, Huu Anh Dang

**Affiliations:** 1 Department of Microbiology and Infectious Diseases, Faculty of Veterinary Medicine, Vietnam National University of Agriculture, Gia Lam, Hanoi 12406, Vietnam.; 2 Key Laboratory of Veterinary Biotechnology, Faculty of Veterinary Medicine, Vietnam National University of Agriculture, Gia Lam, Hanoi 12406, Vietnam.

**Keywords:** avian pathogenic Escherichia coli, chickens, colibacillosis, gene co-occurrence, northern Vietnam, poultry, virulence-associated genes, virulence profiles

## Abstract

**Background and Aim:**

Avian pathogenic *Escherichia coli* (APEC), a major cause of colibacillosis in poultry, possesses diverse virulence-associated genes (VAGs) that contribute to extraintestinal pathogenicity. However, comprehensive information on VAG combinations and co-occurrence patterns among APEC isolates in Vietnam remains limited. This study aimed to characterize VAG prevalence, combination profiles, and co-occurrence patterns in APEC and non-pathogenic *E. coli* (NPEC) isolates from chickens in northern Vietnam.

**Materials and Methods:**

From August 2023 to August 2025, 515 spleen samples were collected from healthy and non-healthy chickens on 35 commercial poultry farms in Hanoi, Hai Phong, Phu Tho, Bac Ninh, and Thai Nguyen. A total of 200 *E. coli* isolates were recovered and screened by multiplex polymerase chain reaction for 19 VAGs: *fimC, eaeA, papC, tsh, sfa_foc, iucD, iutA, irp2, fyuA, iucA, iroN, vat, hlyA, hlyF, astA, ompT, iss, cva/cvi,* and *colV*. Isolates carrying ≥5 of nine selected APEC-associated VAGs were classified as APEC; the remainder were classified as NPEC. We compared gene prevalence, combination profiles, and pairwise co-occurrence using appropriate statistical tests and Jaccard similarity analysis.

**Results:**

Among 200 isolates, 121 (60.5%) were classified as APEC and 79 (39.5%) as NPEC. APEC isolates most frequently carried *hlyF* (100%), *fimC* and *iutA* (96.7% each), *ompT* and *iucD* (95.9% each), *iss* (90.1%), *irp2* (79.3%), and *iroN* (65.3%). APEC isolates contained up to 19 VAGs, whereas NPEC isolates contained up to 14. Twenty-three distinct VAG combination profiles were identified among APEC isolates, and 103/121 (85.1%) harbored >8 VAGs. In contrast, NPEC isolates displayed 13 less complex profiles containing 3–6 VAGs. Most VAGs were significantly more prevalent in APEC than in NPEC isolates, while *papC, fyuA,* and *eaeA* occurred exclusively in APEC. Co-occurrence analyses demonstrated greater complexity and stronger VAG associations among APEC isolates.

**Conclusion:**

APEC isolates from northern Vietnam exhibited extensive VAG diversity and complex co-occurrence patterns distinct from those of NPEC isolates. These findings provide a molecular basis for improved APEC characterization, epidemiological surveillance, and targeted control of poultry colibacillosis.

## INTRODUCTION

Colibacillosis comprises localized or systemic infections caused by avian pathogenic *Escherichia coli* (APEC) [1, 2]. The disease manifests in several forms, including hemorrhagic septicemia, perihepatitis, pericarditis, coligranuloma (Hjarre’s disease), airsacculitis, swollen-head syndrome, venereal colibacillosis, coliform cellulitis, peritonitis, salpingitis, orchitis, and osteomyelitis/synovitis [3, 4]. Colibacillosis causes substantial economic losses to the global poultry industry [5–7]. In the USA, poultry flocks affected by colibacillosis had condemnation rates of 0.23% antemortem and 0.90% postmortem from January to October 2017, with airsacculitis and septicemia accounting for 17.3% and 35% of chicken condemnations, respectively [8].

Based on experimental pathogenicity assessments in chicks, APEC strains have been classified into high-, intermediate-, and low-pathogenicity groups [9, 10]. The pathogenic potential of APEC is largely determined by virulence-associated genes (VAGs) and pathogenicity-associated islands (PAIs) which contribute to disease severity in poultry [1, 11]. VAGs enable APEC to colonize, invade, and survive in extraintestinal environments through multiple mechanisms, including adhesion, toxin production, resistance to host defense mechanisms, iron acquisition, hemolysis, and invasion [1, 2, 9, 12–16]. Several VAGs have been identified as important contributors to APEC pathogenicity, including *papC* (P fimbriae), *iucD* (aerobactin system), *irp2* (iron-repressible protein), *tsh* (temperature-sensitive hemagglutinin), *vat* (vacuolating autotransporter toxin), *astA* (enteroaggregative toxin), *iss* (increased serum survival protein), and the colicin V plasmid operon genes *cva/cvi* [14]. Johnson *et al.* [9] reported that five plasmid-associated VAGs, *hlyF*, *ompT*, *iroN*, *iss*, and *iutA*, were among the most frequently detected genes in APEC strains and were strongly associated with avian pathogenicity.

Numerous VAGs have been identified in APEC, many of which are located within chromosomal or plasmid-associated PAIs [1, 17–19]. For example, studies in eastern China identified up to 15 virulence genes among APEC isolates, including *fimC*, *mat*, *iss*, *ompA*, *ibeB*, *papC*, *irp2*, *fyuA*, *neuC*, *tsh*, *iucD*, *iroN*, *ibeA*, and *vat* [19]. Similarly, long-term surveillance studies in Korea reported frequent detection of *iroN*, *ompT*, *fimC*, *hlyF*, *iss*, *iucD*, *tsh*, *fyuA*, and *irp2* in APEC isolates [18]. Importantly, APEC pathogenicity is not determined by a single gene but rather by combinations of multiple VAGs, with individual isolates typically harboring 2–13 VAGs [10, 18, 20–23]. Previous studies have adopted a threshold of at least five VAGs (≥5 VAGs) because *E. coli* isolates carrying multiple VAGs exhibit greater pathogenic potential and are more frequently associated with systemic colibacillosis [20, 23, 24]. Although the five-marker predictor set proposed by Johnson *et al.* [9] (*iutA*, *hlyF*, *iss*, *iroN*, and *ompT*) is widely used for APEC identification, subsequent studies have classified isolates harboring five or more VAGs as APEC and those carrying fewer than five VAGs as non-pathogenic *E. coli* (NPEC), thereby providing a broader assessment of virulence diversity and pathogenic potential [20, 23, 24].

Poultry production is one of the most important livestock sectors in Vietnam, particularly in the northern provinces, where intensive commercial poultry production is concentrated [25]. Colibacillosis caused by APEC remains a major cause of mortality, reduced productivity, carcass condemnation, and economic losses in poultry production worldwide [26]. Available evidence indicates that APEC isolates from chickens in Vietnam harbor VAGs broadly consistent with global patterns [27–29]. Furthermore, Hanoi, Hai Phong, Phu Tho, Bac Ninh, and Thai Nguyen are major poultry-producing areas with high poultry densities [25], emphasizing the importance of regional surveillance of pathogenic *E. coli* populations.

Despite the importance of colibacillosis in poultry production, comprehensive information on VAG combinations and molecular pathotypes of APEC circulating in Vietnam remains limited [27–29]. Previous Vietnamese studies have primarily focused on antimicrobial resistance, individual VAGs, or characterization of single strains [27–29]. Consequently, large-scale information on VAG combination profiles and co-occurrence patterns among APEC isolates, particularly in direct comparison with NPEC populations, remains scarce. This limitation restricts a comprehensive understanding of the diversity and organization of virulence determinants associated with APEC pathogenicity and epidemiology in Vietnam. In addition, comparative information on the geographical distribution of VAG profiles across major poultry-producing provinces in northern Vietnam remains insufficient. Comprehensive characterization of VAG prevalence, combination profiles, and co-occurrence patterns is therefore needed to strengthen molecular epidemiological surveillance and support targeted strategies for controlling poultry colibacillosis.

This study aimed to comprehensively characterize the prevalence and VAG profiles of APEC isolates recovered from chickens in northern Vietnam. The secondary objectives were to compare VAG profiles and co-occurrence networks between APEC and NPEC isolates and assess their geographical distribution across the study area. The 19 VAGs investigated represented major pathogenic mechanisms associated with APEC infection and included adhesin-related genes (*fimC*, *eaeA*, *papC*, *tsh*, and *sfa_foc*), iron acquisition genes (*iucD*, *iutA*, *irp2*, *fyuA*, *iucA*, and *iroN*), toxin-related genes (*vat*, *hlyA*, *hlyF*, and *astA*), protectin-related genes (*ompT* and *iss*), and other virulence-associated factors (*cva/cvi* and *colV*). These genes are considered important contributors to extraintestinal pathogenicity and systemic colibacillosis [1, 3, 26, 30]. Characterization of their prevalence, combination profiles, and co-occurrence patterns may provide a molecular basis for differentiating APEC from NPEC isolates and support targeted surveillance, prevention, and control strategies for poultry colibacillosis in Vietnam.

## MATERIALS AND METHODS

### Ethical approval

The Animal Ethics Committee of Vietnam National University of Agriculture, Hanoi, Vietnam, reviewed and approved the study protocol (Approval No. VNUA-2023/05; approved on August 10, 2023). Spleen samples were collected from naturally diseased, dead, or clinically healthy chickens during routine veterinary diagnostic investigations and with permission from the respective farm owners. The study did not involve experimental infection, deliberate disease induction, or any additional invasive procedures performed specifically for research purposes. Sample collection, animal handling, and subsequent laboratory investigations were conducted in accordance with the conditions stipulated in the approved study protocol and applicable institutional animal welfare requirements. Only samples obtained during routine diagnostic or veterinary examination procedures were included in the study, thereby minimizing additional handling and potential distress to the animals.

### Study period and location

The study was conducted from August 2023 to August 2025 on 35 commercial poultry farms located in five major poultry-producing provinces of northern Vietnam: Hanoi, Hai Phong, Phu Tho, Bac Ninh, and Thai Nguyen. The selected farms represented both intensive and backyard production systems and included broiler, layer, and breeder flocks of different sizes and age groups.

### Study design

A cross-sectional study was conducted to characterize the distribution, profiles, and co-occurrence patterns of VAGs among APEC and NPEC isolates recovered from chickens. A total of 515 chickens from 35 poultry farms were included. Spleen samples were collected from 365 sick or dead chickens showing clinical signs or postmortem lesions consistent with colibacillosis and 150 clinically healthy chickens without apparent clinical signs or gross pathological lesions at sampling. Isolated *E. coli* strains were screened for 19 VAGs using multiplex polymerase chain reaction (mPCR), and isolates were subsequently classified as APEC or NPEC according to their VAG profiles. The study focused specifically on VAG distribution and co-occurrence; therefore, serotyping, phylogrouping, and multilocus sequence typing analyses were not included in the study design.

### Sample size estimation

The minimum sample size was estimated using the standard formula for cross-sectional prevalence studies described by Naing *et al.* [31]. At the farm-level, an expected APEC prevalence of 90% [32], a 95% confidence level, and 10% precision yielded a minimum required sample size of 35 farms. Accordingly, 35 commercial poultry farms were included. At the individual level, based on the expected APEC prevalence of 20%–30% reported by Kathayat *et al.* [1] and a 95% confidence level, the recommended sample size was 9–14 chickens per farm. Approximately 14–15 chickens were therefore randomly sampled from each farm, exceeding the minimum requirement while maintaining an appropriate balance between statistical power and field feasibility. This resulted in a total sample size of 515 chickens.

### Sample collection

A total of 515 spleen samples were randomly collected from broiler, layer, and breeder flocks on the 35 study farms. The spleen was selected because, as a major component of the reticuloendothelial system, it reflects systemic bacterial dissemination during colisepticemia. Splenomegaly and congestion are recognized postmortem findings associated with acute *E. coli* septicemia in poultry [33], and spleen sampling together with other internal organs is widely used in APEC isolation protocols [1]. Uniform collection of spleen samples from both non-healthy and clinically healthy chickens also reduced the potential for environmental or fecal cross-contamination associated with organs adjacent to the gastrointestinal tract and provided a consistent basis for comparison between APEC and NPEC isolates.

Of the 515 samples, 365 were collected from sick or dead chickens exhibiting clinical signs or characteristic postmortem lesions consistent with colibacillosis, including airsacculitis, fibrinous pericarditis, perihepatitis, septicemia, and splenomegaly. These birds were categorized as non-healthy chickens. The remaining 150 samples were obtained from clinically healthy chickens that showed no apparent clinical signs or gross pathological lesions at the time of sampling.

### Bacterial isolation and identification

Spleen samples were cultured on MacConkey agar (Merck KGaA, Darmstadt, Germany) and incubated at 37°C for 24 h. Bright pink colonies surrounded by precipitate were aseptically subcultured onto eosin methylene blue agar (Merck KGaA) and incubated at 37°C for 24 h. Single colonies with a greenish-black appearance and metallic sheen were selected for biochemical characterization using the API 20E kit (bioMérieux, Marcy-l’étoile, France) according to the manufacturer’s instructions.

Presumptive isolates were subsequently confirmed as *E. coli* by PCR using primer pairs described by Bej *et al.* [34]. All confirmed isolates were stored in brain heart infusion broth (Merck KGaA) supplemented with 20% glycerol at −80°C until further analysis.

### DNA extraction

DNA was extracted from *E. coli* isolates using the boiling–freeze-thaw method [10, 35, 36]. Briefly, isolates were cultured on nutrient agar (Merck KGaA) at 37°C for 24 h. Smooth, circular, white colonies were suspended in 200 µL of deionized water, thoroughly vortexed, and boiled at 100°C for 20 min. The bacterial suspensions were subsequently cooled on ice for 20 min and centrifuged at 14,000 x *g* for 10 min at 4°C. The supernatant containing bacterial DNA was transferred to a new sterile microcentrifuge tube, adjusted to 100 ng/µL, and stored at −20°C until mPCR analysis.

### Detection of VAGs by multiplex PCR

The mPCR assay was used to detect 19 VAGs in the *E. coli* isolates. Each reaction contained DNA at 100 ng/µL, 10 pmol of each primer pair (Integrated DNA Technologies, Coralville, IA, USA), 10× PCR buffer (Sigma-Aldrich, St. Louis, MO, USA), magnesium chloride (MgCl₂) (50 mM), 2.5 U of Taq DNA polymerase (Quantabio, Beverly, USA), deoxyribonucleotide triphosphates (dNTPs) (Invitrogen, Carlsbad, CA, USA), and PCR-grade water. The total reaction volume was 25 µL and consisted of 5 µL of DNA, 1 µL of each primer, 2.5 µL of 10× PCR buffer, 1.25 µL of MgCl₂, 1 µL of dNTPs, 0.5 µL of Taq DNA polymerase, and PCR-grade water to a final volume of 25 µL, according to the corresponding multiplex group presented in Table 1.

Thermal cycling consisted of an initial denaturation at 94°C for 3 min 30 s, followed by 35 cycles of denaturation at 94°C for 30 s, annealing for 1 min at the temperature specified for each multiplex group (Tm1 = 56°C, Tm2 = 57°C, Tm3 = 54°C, Tm4 = 58°C, and Tm5 = 56°C; Table 1), and extension at 72°C for 1 min 30 s. A final extension was performed at 72°C for 6 min, after which the products were maintained at 4°C.

The mPCR products were separated by electrophoresis on 1% agarose gels containing ethidium bromide together with a 100-bp DNA ladder (Cleaver Scientific, Rugby, UK) for 30 min and visualized under ultraviolet illumination using a gel documentation system (Bio-Rad Laboratories, Inc., Hercules, CA, USA). A reaction was considered positive when a distinct band corresponding to the expected amplicon size was observed relative to the 100-bp DNA ladder. Because amplification results were interpreted based on discrete DNA fragments of predetermined sizes, dedicated gel image-analysis software was not required.

All primers were obtained from previously published and validated studies and were commercially synthesized. Primer specificity and expected amplicon sizes were verified according to the original publications and further confirmed in the present study using a previously characterized APEC-positive-control strain. Each multiplex group included an APEC reference strain carrying the corresponding target VAGs as a positive-control and nuclease-free water as a negative control. The positive-control strain was isolated from chickens and maintained at the Key Laboratory of Veterinary Biotechnology, Faculty of Veterinary Medicine, Vietnam National University of Agriculture. Primer sequences, expected amplicon sizes, multiplex groups, and corresponding annealing temperatures are presented in Table 1[9, 10, 12, 14, 37–39].

### Classification of APEC and NPEC isolates

Classification of *E. coli* isolates as APEC or NPEC was based on the VAG profiles determined by mPCR. Isolates harboring five or more VAGs (≥5 VAGs) were classified as APEC, whereas isolates carrying fewer than five VAGs were classified as NPEC, following previously reported criteria [20, 23, 24]. This threshold has been applied because isolates harboring multiple VAGs exhibit greater pathogenic potential and are more frequently associated with systemic colibacillosis.

Johnson *et al.* [9] identified five minimal predictor genes (*iutA*, *hlyF*, *iss*, *iroN*, and *ompT*) strongly associated with APEC pathogenicity. Challenge experiments in 1-day-old chicks further demonstrated that isolates harboring fewer than two of these five marker genes rarely exhibited pathogenicity, whereas most virulent APEC strains possessed two or more [40]. Furthermore, Johar *et al.* [23] classified chicken *E. coli* isolates harboring at least five of the investigated VAGs (*ompT*, *hlyF*, *iroN*, *iucD*, *iss*, *tsh*, *vat*, and *cva/cvi*) as APEC, whereas isolates carrying fewer than five genes were classified as NPEC.

Based on these previously validated criteria, nine selected APEC-associated VAGs (*ompT, hlyF, iroN, iutA, tsh, vat, iss, cva/cvi*, and *iucD*) were used for APEC classification in the present study. An *E. coli* isolate was classified as APEC when it carried ≥5 of these nine selected VAGs, whereas isolates carrying <5 of these nine VAGs were classified as NPEC. A chicken was considered APEC-positive when its *E. coli* isolate carried ≥5 selected APEC-associated VAGs. A farm was considered APEC-positive when at least one sampled chicken was APEC-positive [32].

**Table 1:** Primers used for multiplex polymerase chain reaction detection of virulence-associated genes.

**Group**	**Description**	**Specific gene**	**Primer sequence (5′–3** **′)ᵃ**	**Expected amplicon size (bp)**	**Annealing temperature ** **(°C)**	**Reference**
I	Pesticin receptor of Yersinia	fyuA	F: ACCGTTATCGCCATTCTG R: CTGTGAAGTCTGGGCATTAG	235	56	[10]
	Outer membrane protein intimin	eaeA	EaeAF: GACCCGGCACAAGCATAAGC EaeAR: CCACCTGCAGCAACAAGAGG	384		[37]
	Putative avian hemolysin	hlyF	F: GGCCACAGTCGTTTAGGGTGCTTACC R: GGCGGTTTAGGCATTCCGATACTCAG	450		[9]
	Salmochelin siderophore receptor	iroN	F: AATCCGGCAAAGAGACGAACCGCCT R: GTTCGGGCAACCCCTGCTTTGACTTT	553		[9]
	Temperature-sensitive hemagglutinin	tsh	F: ACTATTCTCTGCAGGAAGTC R: CTTCCGATGTTCTGAACGT	824		[38]
II	Aerobactin production	iucA	F: CGCGAGCGGCTCATACAGG R: TCGTCGGGCAGCGTTTCT	236	57	[10]
	Increased serum survival	iss	F: CAGCAACCCGAACCACTTGATG R: AGCATTGCCAGAGCGGCAGAA	323		[9]
	Iron-repressible protein	irp2	F: AAGGATTCGCTGTTACCGGAC R: AACTCCTGATACAGGTGGC	413		[14]
	Colicin V plasmid operon	cva/cvi	F: TGGTAGAATGTGCCAGAGCAAG R: GAGCTGTTTGTAGCGAAGCC	1181		[14]
III	Hemolysin operon transport gene	hlyA	F: TTGGGATACGCTGATAGG R: CACCCTTGACTAATAACTCG	290	54	[10]
	Type 1 fimbrial adhesin	fimC	F: GCCGATGGTGTAAAGGAT R: CCGTCAGGTAATAGGGTGT	337		[10]
	Episomal outer membrane protease	ompT	F: TCATCCCGGAAGCCTCCCTCACTACTAT R: TAGCGTTTGCTGCACTGGCTTCTGATAC	496		[9]
	Vacuolating autotransporter toxin	vat	F: TAAATGAGGTGGGCTGTG R: AGGATGCCTCCGTAAACT	861		[10]
IV	Colicin V production	colV	F: ACGGATGCTCAGTTTCT R: TGTGCTTGGCGTCATAG	307	58	[10]
	P pilus-associated gene	papC	F: TGA TAT CAC GCA GTC AGT AG R: CCG GCC ATA TTC ACA TAA	501		[9]
	Aerobactin synthesis gene	iucD	F: ACAAAAAGTTCTATCGCTTCC R: CCTGATCCAGATGATGCTC	714		[12]
V	Enteroaggregative heat-stable toxin	astA	F: TGC CAT CAA CAC AGT ATA TC R: TCA GGT CGC GAG TGA CGGC	116	56	[12]
	Ferric aerobactin receptor	iutA	F: GGC TGG ACA TCA TGG GAA CTGG R: CGT CGG GAA CGG GTA GAA TCG	302		[9]
	S fimbriae	sfa_foc	Sfa1: CTCCGGAGAACTGGGTGCATCTTAC Sfa2: CGGAGGAGTAATTACAAACCTGGCA	410		[39]

ᵃF: Forward; R: reverse. Ref.: References. Amplicon sizes corresponded to the gel bands shown in Figure 1.

### Statistical analysis

Statistical analyses were performed using R software (version 4.3.0; R Foundation for Statistical Computing, Vienna, Austria). The prevalence of each VAG was expressed as a percentage with a 95% confidence interval (CI), calculated using the Clopper–Pearson exact binomial method. Differences in the prevalence of individual VAGs between groups were assessed using Pearson’s chi-square test or Fisher’s exact test, as appropriate. All tests were two-sided, and p < 0.05 was considered statistically significant.

Pairwise co-occurrence of VAGs was quantified using the Jaccard similarity coefficient based on gene presence/absence data. The Jaccard similarity coefficient was selected because the analysis focused specifically on the co-occurrence of gene presence, and several VAGs had very high prevalence, making correlation-based measures such as the phi coefficient less informative. Co-occurrence patterns were visualized using hierarchical clustering heatmaps and network analysis, with edge thickness proportional to the Jaccard similarity coefficient. UpSet plots were generated to visualize the frequency and complexity of VAG combination patterns in APEC and NPEC isolates.

## RESULTS

### Isolation of *E. coli* from chicken spleen samples

A total of 160 *E. coli* isolates were recovered from 365 spleen samples collected from non-healthy chickens, corresponding to an isolation rate of 43.84%. Among the 150 spleen samples collected from healthy chickens, 40 (26.67%) were positive for *E. coli*. Overall, *E. coli* was isolated from 200 of 515 samples (38.83%), and all 35 surveyed farms (100%) had at least one *E. coli*-positive sample. The isolation rate was significantly higher in non-healthy chickens than in healthy chickens (p < 0.001). All 200 isolates were subsequently subjected to mPCR for VAG detection.

**Figure 1:**
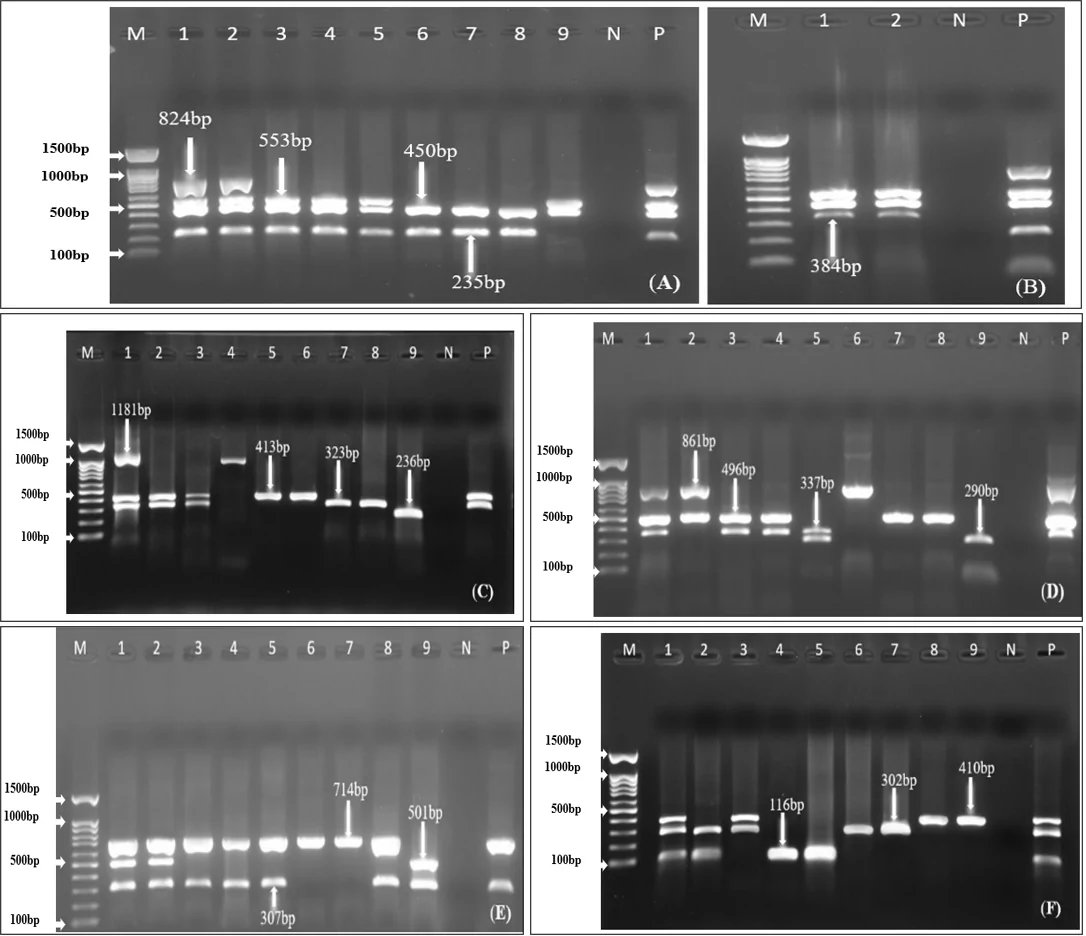
Detection of VAGs by mPCR. Lane M = 100-bp DNA ladder; lane N = Negative control; lane P = Positive-control. (A and B) Detection of *fyuA* (235 bp), *eaeA* (384 bp), *hlyF* (450 bp), *iroN* (553 bp), and *tsh* (824 bp). (C) Detection of *iucA* (236 bp), *iss* (323 bp), *irp2* (413 bp), and *cva/cvi* (1181 bp). (D) Detection of *hlyA* (290 bp), *fimC* (337 bp), *ompT* (496 bp), and *vat* (861 bp). (E) Detection of *colV* (307 bp), *papC* (501 bp), and *iucD* (714 bp). (F) Detection of *astA* (116 bp), *iutA* (302 bp), and *sfa_foc* (410 bp). bp = Base pair; DNA = Deoxyribonucleic acid; mPCR = Multiplex polymerase chain reaction; VAG = Virulence-associated gene.

### Detection of VAGs

All 19 investigated VAGs were detected among the *E. coli* isolates recovered from non-healthy chickens: *fyuA*, *eaeA*, *hlyF*, *iroN*, *tsh*, *iucA*, *iss*, *irp2*, *cva/cvi*, *hlyA*, *fimC*, *ompT*, *vat*, *colV*, *papC*, *iucD*, *astA*, *iutA*, and *sfa_foc*. In contrast, 15 of the 19 VAGs were detected among isolates from healthy chickens; *papC*, *eaeA*, *sfa_foc*, and *hlyA* were not detected in this group (Tables 2, 4, and 5 and Figures 1 and 2).

**Table 2:** Prevalence of virulence-associated genes among Escherichia coli isolates from non-healthy and healthy chickens.

**VAG**	**Non-healthy chickens (n = 160), positive isolates, n**	**Prevalence, % ** **(95% CI)**	**Healthy chickens (n = 40), positive isolates, n**	**Prevalence, % ** **(95% CI)**	**p-value**
fimC	156	97.5 (93.7–99.3)	30	75.0 (58.8–87.3)	<0.0001
ompT	146	91.3 (85.8–95.1)	26	65.0 (48.3–79.4)	<0.001
iutA	145	90.6 (85.0–94.7)	25	62.5 (45.8–77.3)	<0.0001
hlyF	125	78.1 (70.9–84.3)	13	32.5 (18.6–49.1)	<0.0001
iucD	120	75.0 (67.6–81.5)	10	25.0 (12.7–41.2)	<0.0001
iss	112	70.0 (62.3–77.0)	10	25.0 (12.7–41.2)	<0.0001
iroN	100	62.5 (54.5–70.0)	10	25.0 (12.7–41.2)	<0.0001
irp2	98	61.3 (53.2–68.8)	7	17.5 (7.3–32.8)	<0.0001
fyuA	67	41.9 (34.1–49.9)	2	5.0 (0.6–16.9)	<0.0001
vat	59	36.9 (29.4–44.9)	4	10.0 (2.8–23.7)	0.000988
cva/cvi	51	31.9 (24.7–39.7)	10	25.0 (12.7–41.2)	0.448
iucA	50	31.3 (24.2–39.0)	11	27.5 (14.6–43.9)	0.705
astA	47	29.4 (22.4–37.1)	5	12.5 (4.2–26.8)	0.0423
papC	46	28.8 (21.9–36.4)	0	0.0 (0–8.8)	0.000011
colV	43	26.9 (20.2–34.4)	6	15.0 (5.7–29.8)	0.151
tsh	36	22.5 (16.3–29.8)	5	12.5 (4.2–26.8)	0.193
eaeA	26	16.3 (10.9–22.9)	0	0.0 (0–8.8)	0.00298
sfa_foc	19	11.9 (7.3–17.9)	0	0.0 (0–8.8)	0.0158
hlyA	5	3.1 (1.0–7.1)	0	0.0 (0–8.8)	0.585

CI: Confidence interval; VAG: virulence-associated gene.

**Figure 2:**
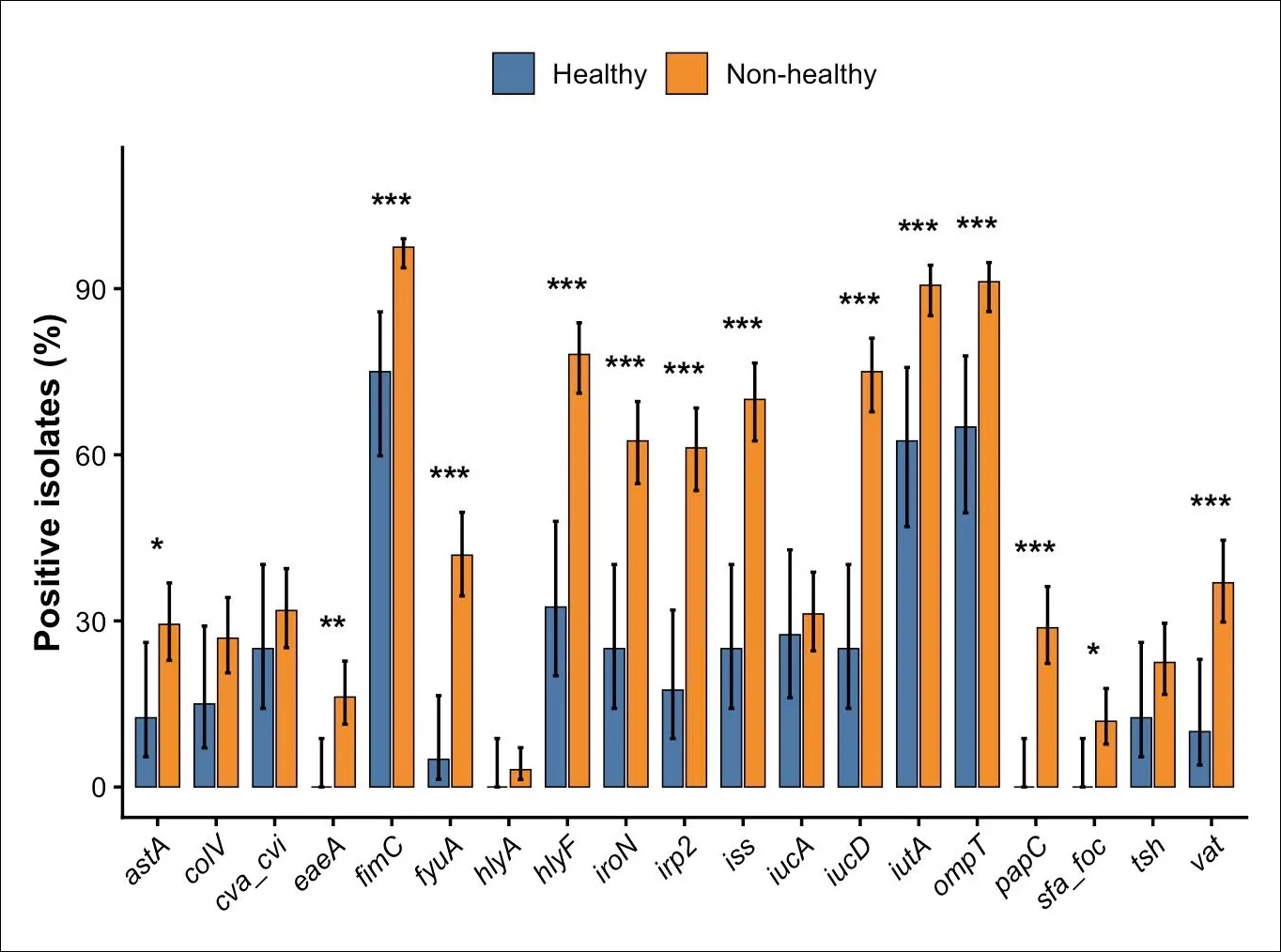
Prevalence of VAGs among *Escherichia coli* isolates recovered from non-healthy and healthy chickens. Bars represent the percentage of positive isolates, and error bars represent 95% CIs. p values indicate comparisons of VAG prevalence between the two groups. CI = Confidence interval; VAG = Virulence-associated gene.

### Distribution of VAGs in *E. coli* isolates from non-healthy and healthy chickens

Among the 160 *E. coli* isolates recovered from non-healthy chickens, *fimC* was the most prevalent VAG, detected in 97.5% of isolates (95% CI: 93.7–99.3), followed by *ompT* (91.3%; 95% CI: 85.8–95.1), *iutA* (90.6%; 95% CI: 85.0–94.7), *hlyF* (78.1%; 95% CI: 70.9–84.3), *iucD* (75.0%; 95% CI: 67.6–81.5), *iss* (70.0%; 95% CI: 62.3–77.0), *iroN* (62.5%; 95% CI: 54.5–70.0), and *irp2* (61.3%; 95% CI: 53.2–68.8). The *tsh*, *colV*, *papC*, *astA*, *iucA*, *cva/cvi*, *vat*, and *fyuA* genes were detected in 22.5%–41.9% of isolates. In contrast, *eaeA* (16.3%; 95% CI: 10.9–22.9), *sfa_foc* (11.9%; 95% CI: 7.3–17.9), and *hlyA* (3.1%; 95% CI: 1.0–7.1) were detected at relatively low frequencies (Table 2 and Figure 2).

Among the 40 *E. coli* isolates recovered from healthy chickens, *fimC* was the most prevalent VAG, detected in 75.0% of isolates (95% CI: 58.8–87.3), followed by *ompT* (65.0%; 95% CI: 48.3–79.4), *iutA* (62.5%; 95% CI: 45.8–77.3), *hlyF* (32.5%; 95% CI: 18.6–49.1), *iucA* (27.5%; 95% CI: 14.6–43.9), and *iucD*, *iss*, *iroN*, and *cva/cvi* (25.0% each; 95% CI: 12.7–41.2). The remaining VAGs were detected at lower frequencies: *fyuA*, 5.0% (95% CI: 0.6–16.9); *vat*, 10.0% (95% CI: 2.8–23.7); *astA*, 12.5% (95% CI: 4.2–26.8); *tsh*, 12.5% (95% CI: 4.2–26.8); *colV*, 15.0% (95% CI: 5.7–29.8); and *irp2*, 17.5% (95% CI: 7.3–32.8). Notably, *papC*, *eaeA*, *sfa_foc*, and *hlyA* were not detected in isolates from healthy chickens (Table 2 and Figure 2).

Comparison between the two groups showed that several VAGs were significantly more prevalent among isolates from non-healthy chickens. Significant differences were observed for *fimC*, *ompT*, *iutA*, *hlyF*, *iucD*, *iss*, *iroN*, *irp2*, and *fyuA* (p < 0.001), as well as for *vat*, *astA*, *papC*, *eaeA*, and *sfa_foc* (p < 0.05). In contrast, the prevalence of *cva/cvi*, *iucA*, *colV*, *tsh*, and *hlyA* did not differ significantly between isolates from non-healthy and healthy chickens (p > 0.05) (Table 2 and Figure 2).

**Table 3:** Prevalence of virulence-associated genes among avian pathogenic Escherichia coli and non-pathogenic Escherichia coli isolates

**VAG**	**APEC isolates (n = 121), positive isolates, n**	**Prevalence, %** **(95% CI)**	**NPEC isolates (n = 79),** **positive isolates, n**	**Prevalence, % ** **(95% CI)**	**p-value**
fimC	117	96.7 (91.8–99.1)	69	87.3 (78.0–93.8)	0.02014
ompT	116	95.9 (90.6–98.6)	56	70.9 (59.6–80.6)	<0.0001
iutA	117	96.7 (91.8–99.1)	53	67.1 (55.6–77.3)	<0.0001
hlyF	121	100.0 (97.0–100.0)	17	21.5 (13.1–32.2)	<0.0001
iucD	116	95.9 (90.6–98.6)	14	17.7 (10.0–27.9)	<0.0001
iss	109	90.1 (83.3–94.8)	13	16.5 (9.1–26.5)	<0.0001
iroN	79	65.3 (56.1–73.7)	31	39.2 (28.4–50.9)	0.000451
irp2	96	79.3 (71.0–86.2)	9	11.4 (5.3–20.5)	<0.0001
fyuA	69	57.0 (47.7–66.0)	0	0.0 (0–4.6)	<0.0001
vat	59	48.8 (39.6–58.0)	4	5.1 (1.4–12.5)	<0.0001
cva/cvi	49	40.5 (31.7–49.8)	12	15.2 (8.1–25.0)	0.000144
iucA	42	34.7 (26.3–43.9)	19	24.1 (15.1–35.0)	0.119
astA	44	36.4 (27.8–45.6)	8	10.1 (4.5–19.0)	<0.0001
papC	46	38.0 (29.3–47.3)	0	0.0 (0–4.6)	<0.0001
colV	38	31.4 (23.3–40.5)	11	13.9 (7.2–23.5)	0.00674
tsh	37	30.6 (22.5–39.6)	4	5.1 (1.4–12.5)	<0.0001
eaeA	26	21.5 (14.5–29.9)	0	0.0 (0–4.6)	<0.0001
sfa_foc	19	15.7 (9.7–23.4)	0	0.0 (0–4.6)	<0.0001
hlyA	5	4.1 (1.4–9.4)	0	0.0 (0–4.6)	0.159

APEC = Avian pathogenic *Escherichia coli*; CI = confidence interval; NPEC = non-pathogenic *Escherichia coli*; VAG = virulence-associated gene.

**Figure 3:**
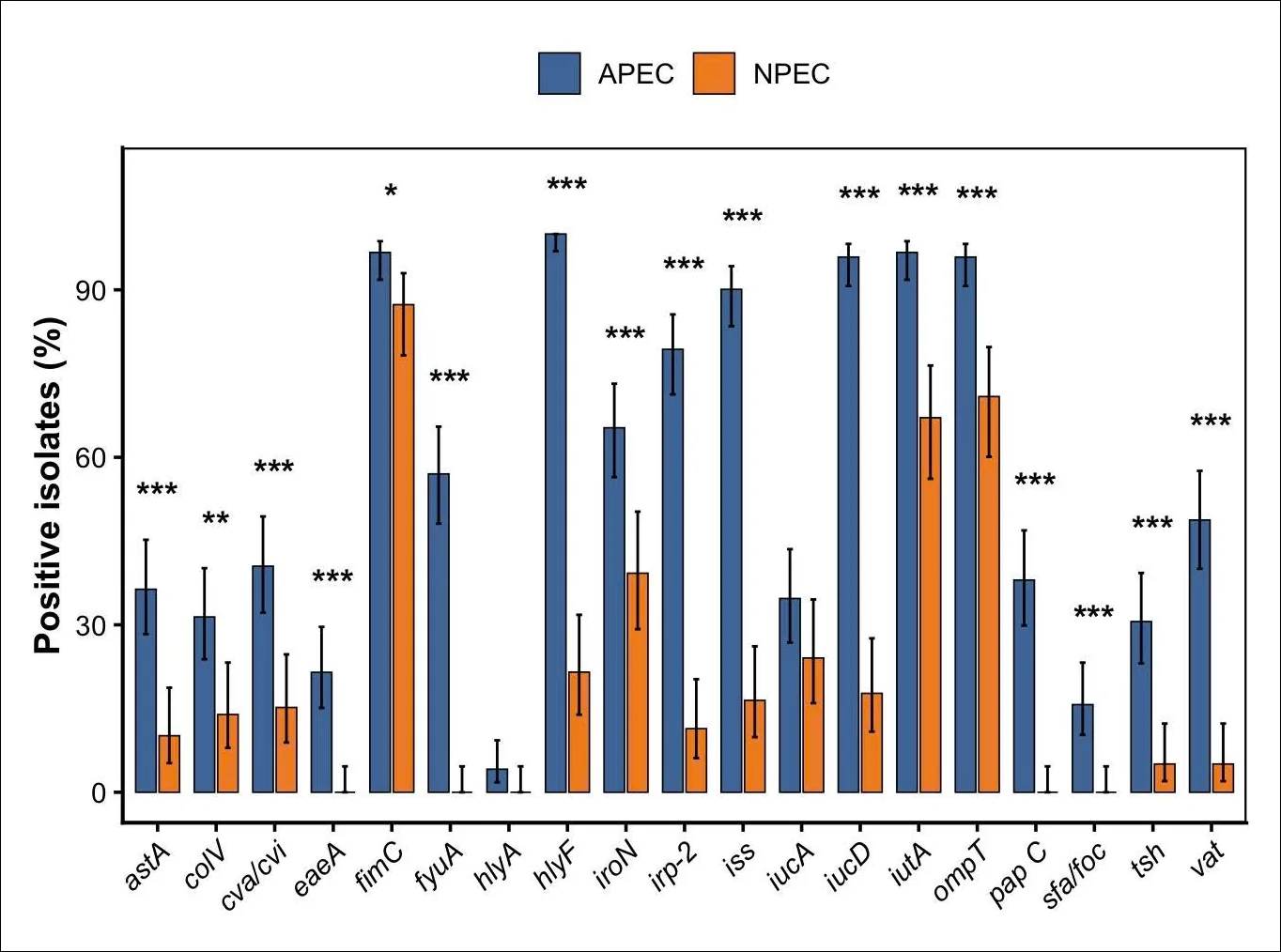
Prevalence of VAGs among APEC and NPEC isolates. Bars represent the percentage of isolates positive for each VAG, and error bars represent 95% CIs. p values indicate comparisons of VAG prevalence between APEC and NPEC isolates. APEC = Avian pathogenic *Escherichia coli*; CI = Confidence interval; NPEC = Non-pathogenic *Escherichia coli*; VAG = Virulence-associated gene.

### Classification and distribution of VAGs in APEC and NPEC isolates

Based on the presence of the selected VAGs *ompT*, *hlyF*, *iroN*, *iutA*, *tsh*, *vat*, *iss*, *cva/cvi*, and *iucD*, 121 of the 200 *E. coli* isolates (60.5%) carrying five or more VAGs were classified as APEC. Among these, 115/121 (95.0%) were recovered from non-healthy chickens, whereas 6/121 (5.0%) were recovered from healthy chickens. The remaining 79/200 isolates (39.5%), which harbored fewer than five VAGs, were classified as NPEC; 45/79 (57.0%) were recovered from non-healthy chickens and 34/79 (43.0%) from healthy chickens (Tables 4 and 5; Figure 3). In addition, 34 of the 35 surveyed farms (97.1%) had at least one APEC-positive sample.

The respective prevalence of VAGs in APEC and NPEC isolates was as follows: *hlyF*, 100.0% (95% CI: 97.0–100.0) and 21.5% (95% CI: 13.1–32.2); *iutA*, 96.7% (95% CI: 91.8–99.1) and 67.1% (95% CI: 55.6–77.3); *ompT*, 95.9% (95% CI: 90.6–98.6) and 70.9% (95% CI: 59.6–80.6); *iucD*, 95.9% (95% CI: 90.6–98.6) and 17.7% (95% CI: 10.0–27.9); *iss*, 90.1% (95% CI: 83.3–94.8) and 16.5% (95% CI: 9.1–26.5); *irp2*, 79.3% (95% CI: 71.0–86.2) and 11.4% (95% CI: 5.3–20.5); *fyuA*, 57.0% (95% CI: 47.7–66.0) and 0% (95% CI: 0–4.6); *vat*, 48.8% (95% CI: 39.6–58.0) and 5.1% (95% CI: 1.4–12.5); *papC*, 38.0% (95% CI: 29.3–47.3) and 0% (95% CI: 0–4.6); *astA*, 36.4% (95% CI: 27.8–45.6) and 10.1% (95% CI: 4.5–19.0); *tsh*, 30.6% (95% CI: 22.5–39.6) and 5.1% (95% CI: 1.4–12.5); *eaeA*, 21.5% (95% CI: 14.5–29.9) and 0% (95% CI: 0–4.6); and *sfa_foc*, 15.7% (95% CI: 9.7–23.4) and 0% (95% CI: 0–4.6). The prevalence of these genes was significantly higher in APEC than in NPEC isolates (p < 0.0001).

The prevalence of iroN [65.3% (95% CI: 56.1–73.7) vs. 39.2% (95% CI: 28.4–50.9)], *cva/cvi* [40.5% (95% CI: 31.7–49.8) vs. 15.2% (95% CI: 8.1–25.0)], and *colV* [31.4% (95% CI: 23.3–40.5) vs. 13.9% (95% CI: 7.2–23.5)] was also significantly higher in APEC than in NPEC isolates (p < 0.01). Similarly, *fimC* was significantly more prevalent in APEC isolates [96.7% (95% CI: 91.8–99.1)] than in NPEC isolates [87.3% (95% CI: 78.0–93.8)] (p < 0.05). In contrast, the prevalence of *iucA* [34.7% (95% CI: 26.3–43.9) vs. 24.1% (95% CI: 15.1–35.0)] and *hlyA* [4.1% (95% CI: 1.4–9.4) vs. 0% (95% CI: 0–4.6)] did not differ significantly between the two groups (p > 0.05) (Table 3 and Figure 3).

The five-marker predictor set described by Johnson *et al.* [9], comprising *hlyF*, *ompT*, *iroN*, *iss*, and *iutA*, also differed markedly between APEC and NPEC isolates. Among APEC isolates, *hlyF* was detected in all isolates (100.0%), followed by *iutA* (96.7%), *ompT* (95.9%), *iss* (90.1%), and *iroN* (65.3%). In NPEC isolates, the corresponding prevalence was substantially lower for *ompT* (70.9%), *iutA* (67.1%), *iroN* (39.2%), *hlyF* (21.5%), and *iss* (16.5%) (p < 0.0001). Simultaneous carriage of all five predictor genes was detected in 70/121 (57.8%) APEC isolates but in none of the 79 NPEC isolates.

### Combinations of VAGs in *Escherichia coli* isolates

Among the 160 *E. coli* isolates recovered from non-healthy chickens, 33 distinct VAG combination patterns containing 3–15 genes were identified (Table 4). Isolates harboring more than eight VAGs accounted for 103/160 (64.37%) and were distributed among 18 combination patterns. Notably, 8/160 isolates (5.0%) simultaneously harbored 15 VAGs: *ompT-iutA-fimC-iucD-hlyF-iss-iroN-irp2-vat-papC-cva/cvi-iucA-colV-eaeA-sfa_foc*.

A further 15/160 isolates (9.4%) simultaneously harbored 14 VAGs and were distributed among three combination patterns: *ompT-iutA-fimC-iucD-hlyF-iss-iroN-irp2-fyuA-vat-papC-iucA-colV-tsh*; *iutA-fimC-iucD-hlyF-iss-iroN-irp2-fyuA-papC-cva/cvi-astA-iucA-colV-hlyA*; and *ompT-iutA-fimC-iucD-hlyF-iss-iroN-irp2-vat-papC-astA-iucA-colV-eaeA*. The remaining 57/160 isolates (35.63%), which harbored 3–7 VAGs, were distributed among 15 combination patterns. The 40 *E. coli* isolates recovered from healthy chickens were classified into nine different combination patterns containing 3–7 VAGs (Table 5). None of the isolates harbored all 19 VAGs simultaneously.

Among isolates from non-healthy chickens, those with combination patterns containing 6–15 VAGs (molecular pathotypes VAG1–VAG22) were classified as APEC, whereas those carrying 3–6 VAGs (VAG23–VAG33) were classified as NPEC (Table 4). Among isolates from healthy chickens, isolates with a five-gene combination (*ompT-iucD-hlyF-vat-cva/cvi*; VAG34) and a seven-gene combination (*ompT-iutA-fimC-iucD-hlyF-fyuA-tsh*; VAG35) were classified as APEC, whereas those harboring 3–6 VAGs (VAG36–VAG42) were classified as NPEC (Table 5). NPEC isolates harbored 3–6 VAGs in total across the 19 VAGs investigated; however, none carried ≥5 of the nine predefined APEC-associated VAGs used for classification. Overall, 23 distinct VAG combination patterns were identified among APEC isolates, compared with 13 among NPEC isolates (Figures 4 and 5).

### Co-occurrence patterns of VAGs

The co-occurrence heatmap showed a denser and more structured pattern of VAG associations among APEC isolates than among NPEC isolates, particularly for genes associated with iron acquisition, serum resistance, and ColV plasmid-associated determinants (Figure 4). In contrast, NPEC isolates exhibited fewer and less extensive co-occurrence associations. Strong associations were observed among *irp2*, *iss*, *hlyF*, *iutA*, *fimC*, *ompT*, and *iucD* in APEC isolates. A distinct cluster was also observed between *iucA* and *colV* (Figure 4).

The genes *eaeA*, *sfa_foc*, and *hlyA* were detected in a subset of APEC isolates and co-occurred with the plasmid-associated determinants *iroN*, *iss*, and *hlyF*. Overall, APEC isolates displayed more numerous and complex VAG co-occurrence patterns than NPEC isolates (Figures 4 and 5).

**Figure 4:**
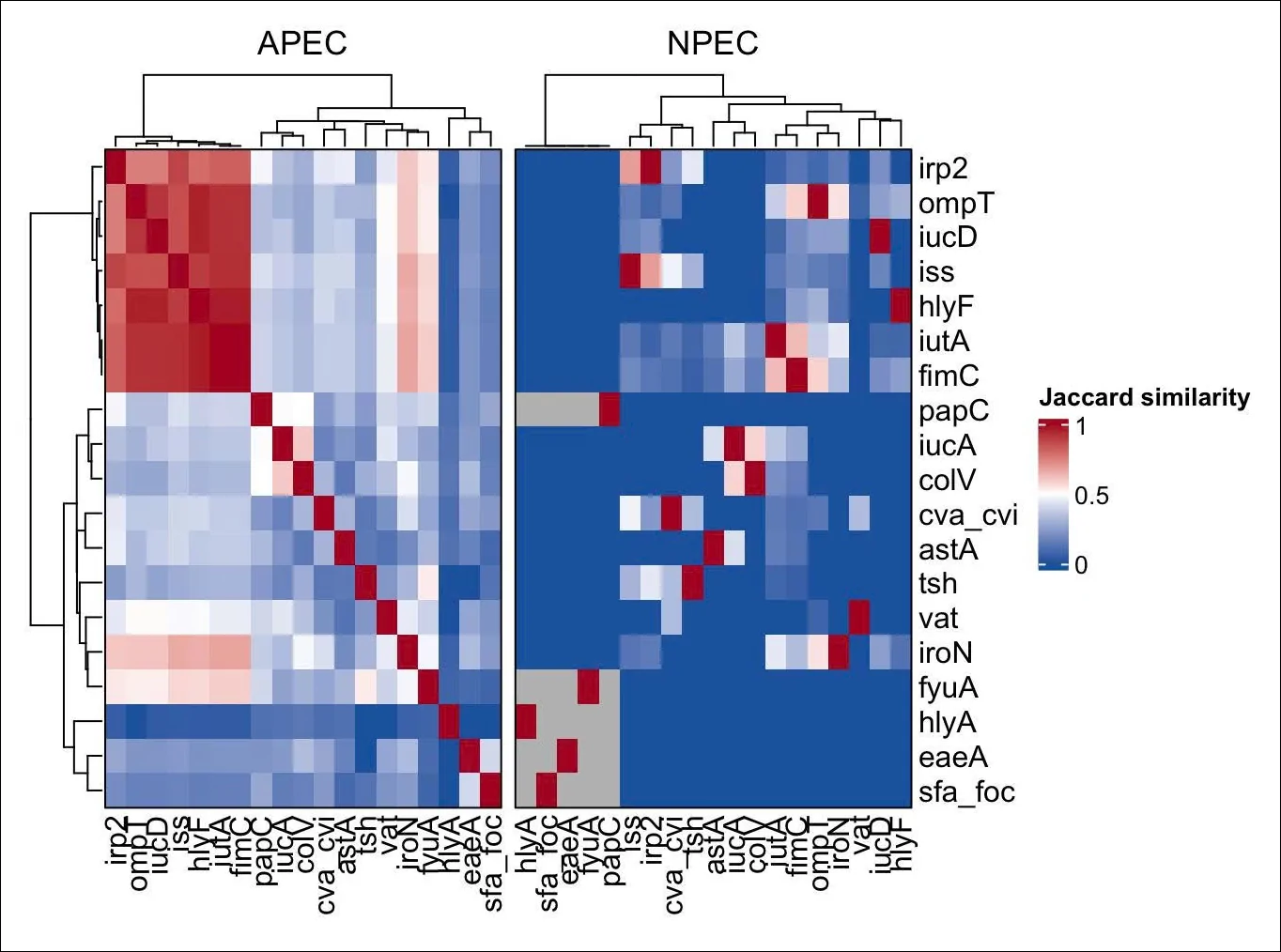
Jaccard similarity heatmaps showing the co-occurrence patterns of 19 VAGs in APEC and NPEC isolates recovered from chickens in northern Vietnam. Hierarchical clustering was performed using the average linkage method based on Jaccard similarity coefficients. Warmer colors (red) indicate stronger co-occurrence between gene pairs, whereas cooler colors (blue) indicate weak or no association. APEC = Avian pathogenic *Escherichia coli*; NPEC = Non-pathogenic *Escherichia coli*; VAG = Virulence-associated gene.

**Figure 5:**
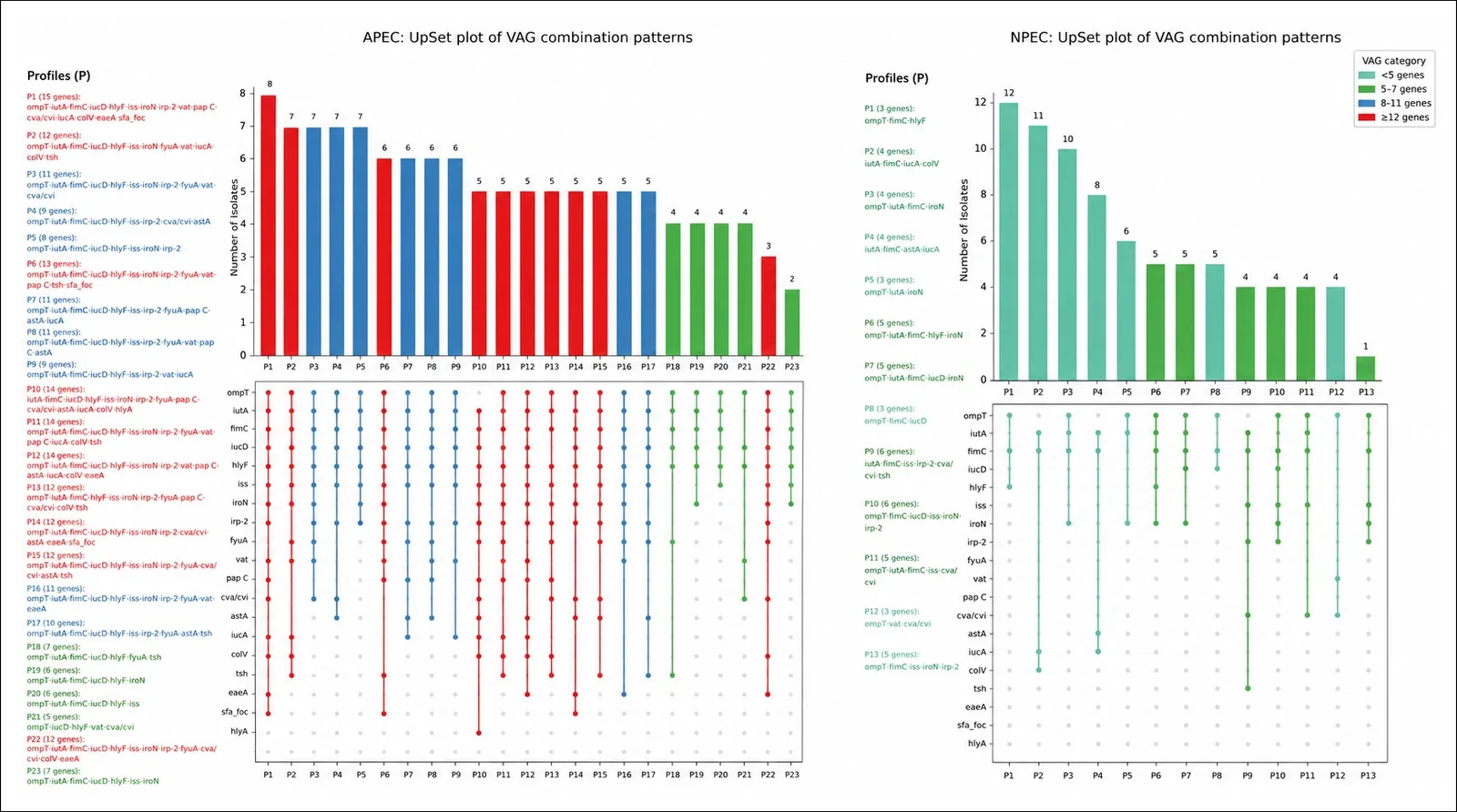
UpSet plots showing the distribution and combination patterns of VAG profiles among APEC (A) and NPEC (B) isolates recovered from chickens in northern Vietnam. The upper bar plots indicate the number of isolates sharing the same VAG profile, whereas the dot matrix below represents the composition of each gene combination. Each profile (P1–P23 for APEC and P1–P13 for NPEC) corresponds to a unique combination of VAGs. Colors indicate the number of VAGs within each profile: green (<5 and 5–7 genes), blue (8–11 genes), and red (≥12 genes). APEC = Avian pathogenic *Escherichia coli*; NPEC = Non-pathogenic *Escherichia coli*; P = Profile; VAG = Virulence-associated gene.

### Distribution of VAG profiles

A total of 23 distinct VAG profiles were identified among the APEC isolates. Most isolates (103/121, 85.1%) were distributed among 18 profiles containing 8–15 VAGs (Tables 4 and 5; Figure 5). The most prevalent profile, P1, comprised 15 VAGs and was detected in eight isolates. This profile included multiple Extraintestinal pathogenic *E. coli *(ExPEC)-associated virulence determinants, namely *ompT*, *iutA*, *fimC*, *iucD*, *hlyF*, *iss*, *iroN*, *irp2*, *fyuA*, *papC*, *cva/cvi*, *iucA*, *colV*, *eaeA*, and *sfa_foc*. Isolates carrying this profile were recovered from diseased chickens exhibiting characteristic gross pathological lesions, including perihepatitis, pericarditis, airsacculitis, swollen-head syndrome, salpingitis, osteomyelitis/synovitis, and markedly enlarged and hemorrhagic spleens. The remaining 18 APEC isolates (14.9%) were distributed among profiles containing 5–7 VAGs.

In contrast, 13 distinct VAG profiles were identified among NPEC isolates, with most profiles comprising 3–6 VAGs (Tables 4 and 5; Figure 5). The predominant NPEC profiles mainly included *ompT*, *fimC*, *iutA*, *hlyF*, and *iroN*, whereas *papC*, *fyuA*, *eaeA*, *sfa_foc*, and *hlyA* were detected infrequently. Overall, APEC isolates exhibited greater diversity and complexity of VAG combinations than NPEC isolates.

The predominance of APEC profiles containing numerous ExPEC-associated virulence determinants indicates a broader virulence gene repertoire than that observed among NPEC isolates. However, because pathogenicity was not confirmed by experimental challenge in this study, these molecular profiles should be interpreted as indicators of virulence-associated potential rather than definitive evidence of pathogenicity.

Among the APEC isolates, the most prevalent VAG profile was *ompT-iutA-fimC-iucD-hlyF-iss-iroN-irp2-vat-papC-cva/cvi-iucA-colV-eaeA-sfa_foc*, which was detected in 8/121 isolates (6.6%). In contrast, the predominant VAG profile among NPEC isolates was *ompT-fimC-hlyF*, which was detected in 12/79 isolates (15.2%).

**Table 4:** Combination patterns of virulence-associated genes among Escherichia coli isolates from non-healthy chickens (n = 160).

**Classifi** **-** **cation**	**Molecular pathotype**	**Number ** **of VAGs**	**Positive isolates, n**	**Positive isolates ** **(** **%)**	**ompT**	**iutA**	**fimC**	**iucD**	**hlyF**	**iss**	**iroN**	**irp2**	**fyuA**	**vat**	**papC**	**cva** **/cvi**	**astA**	**iucA**	**colV**	**tsh**	**eaeA**	**sfa** **_** **foc**	**hlyA**
APEC	VAG 1	15	8	5.0	+	+	+	+	+	+	+	+	−	+	+	+	−	+	+	−	+	+	−
	VAG 2	14	5	3.1	+	+	+	+	+	+	+	+	+	+	+	−	−	+	+	+	−	−	−
	VAG 3	14	5	3.1	−	+	+	+	+	+	+	+	+	−	+	+	+	+	+	−	−	−	+
	VAG 4	14	5	3.1	+	+	+	+	+	+	+	+	−	+	+	−	+	+	+	−	+	−	−
	VAG 5	13	6	3.8	+	+	+	+	+	+	+	+	+	+	+	−	−	−	−	+	−	+	−
	VAG 6	12	5	3.1	+	+	+	−	+	+	+	+	+	−	+	+	−	−	+	+	−	−	−
	VAG 7	12	5	3.1	+	+	+	+	+	+	+	+	+	−	−	+	+	−	−	+	−	−	−
	VAG 8	12	7	4.4	+	+	+	+	+	+	+	−	+	+	−	−	−	+	+	+	−	−	−
	VAG 9	12	3	1.9	+	+	+	+	+	+	+	+	+	−	−	+	−	−	+	−	+	−	−
	VAG 10	12	5	3.1	+	+	+	+	+	+	+	+	−	−	−	+	+	−	−	−	+	+	−
	VAG 11	11	6	3.8	+	+	+	+	+	+	−	+	+	+	+	−	+	−	−	−	−	−	−
	VAG 12	11	5	3.1	+	+	+	+	+	+	+	+	+	+	−	−	−	−	−	−	+	−	−
	VAG 13	11	6	3.8	+	+	+	+	+	+	−	+	+	−	+	−	+	+	−	−	−	−	−
	VAG 14	11	7	4.4	+	+	+	+	+	+	+	+	+	+	−	+	−	−	−	−	−	−	−
	VAG 15	10	5	3.1	+	+	+	+	+	+	−	+	+	−	−	−	+	−	−	+	−	−	−
	VAG 16	9	7	4.4	+	+	+	+	+	+	−	+	−	−	−	+	+	−	−	−	−	−	−
	VAG 17	9	6	3.8	+	+	+	+	+	+	−	+	−	+	−	−	−	+	−	−	−	−	−
	VAG 18	8	7	4.4	+	+	+	+	+	+	+	+	−	−	−	−	−	−	−	−	−	−	−
	VAG 19	7	2	1.3	+	+	+	+	+	−	−	−	+	−	−	−	−	−	−	+	−	−	−
	VAG 20	7	2	1.3	+	+	+	+	+	+	+	−	−	−	−	−	−	−	−	−	−	−	−
	VAG 21	6	4	2.5	+	+	+	+	+	−	+	−	−	−	−	−	−	−	−	−	−	−	−
	VAG 22	6	4	2.5	+	+	+	+	+	+	−	−	−	−	−	−	−	−	−	−	−	−	−
NPEC	VAG 23	6	1	0.6	−	+	+	−	−	+	−	+	−	−	−	+	−	−	−	+	−	−	−
	VAG 24	5	1	0.6	+	−	+	−	−	+	+	+	−	−	−	−	−	−	−	−	−	−	−
	VAG 25	5	1	0.6	+	+	+	−	−	+	−	−	−	−	−	+	−	−	−	−	−	−	−
	VAG 26	5	5	3.1	+	+	+	−	+	−	+	−	−	−	−	−	−	−	−	−	−	−	−
	VAG 27	5	5	3.1	+	+	+	+	−	−	+	−	−	−	−	−	−	−	−	−	−	−	−
	VAG 28	4	5	3.1	−	+	+	−	−	−	−	−	−	−	−	−	−	+	+	−	−	−	−
	VAG 29	4	3	1.9	−	+	+	−	−	−	−	−	−	−	−	−	+	+	−	−	−	−	−
	VAG 30	4	10	6.3	+	+	+	−	−	−	+	−	−	−	−	−	−	−	−	−	−	−	−
	VAG 31	3	4	2.5	+	−	−	−	−	−	−	−	−	+	−	+	−	−	−	−	−	−	−
	VAG 32	3	5	3.1	+	−	+	−	+	−	−	−	−	−	−	−	−	−	−	−	−	−	−
	VAG 33	3	5	3.1	+	−	+	+	−	−	−	−	−	−	−	−	−	−	−	−	−	−	−

APEC = Avian pathogenic *Escherichia coli*; NPEC = Non-pathogenic *Escherichia coli*; VAG = Virulence-associated gene; + = Positive for the corresponding VAG; − = Negative for the corresponding VAG.

**Table 5:** Combination patterns of virulence-associated genes among Escherichia coli isolates from healthy chickens (n = 40).

**Classifi** **-** **cation**	**Molecular pathotype**	**Number ** **of VAGs**	**Positive ** **isolates, n**	**Positive ** **isolates (%)**	**ompT**	**iutA**	**fimC**	**iucD**	**hlyF**	**iss**	**iroN**	**irp2**	**fyuA**	**vat**	**papC**	**cva** **/** **cvi**	**astA**	**iucA**	**colV**	**tsh**	**eaeA**	**sfa** **_** **foc**	**hlyA**
APEC	VAG 34	5	4	10.0	+	−	−	+	+	−	−	−	−	+	−	+	−	−	−	−	−	−	−
	VAG 35	7	2	5.0	+	+	+	+	+	−	−	−	+	−	−	−	−	−	−	+	−	−	−
NPEC	VAG 36	6	3	7.5	−	+	+	−	−	+	−	+	−	−	−	+	−	−	−	+	−	−	−
	VAG 37	6	4	10.0	+	−	+	+	−	+	+	+	−	−	−	−	−	−	−	−	−	−	−
	VAG 38	5	3	7.5	+	+	+	−	−	+	−	−	−	−	−	+	−	−	−	−	−	−	−
	VAG 39	4	6	15.0	−	+	+	−	−	−	−	−	−	−	−	−	−	+	+	−	−	−	−
	VAG 40	4	5	12.5	−	+	+	−	−	−	−	−	−	−	−	−	+	+	−	−	−	−	−
	VAG 41	3	6	15.0	+	+	−	−	−	−	+	−	−	−	−	−	−	−	−	−	−	−	−
	VAG 42	3	7	17.5	+	−	+	−	+	−	−	−	−	−	−	−	−	−	−	−	−	−	−

APEC = Avian pathogenic *Escherichia coli*; NPEC = Non-pathogenic *Escherichia coli*; VAG = Virulence-associated gene; + = Positive for the corresponding VAG; − = Negative for the corresponding VAG.

## DISCUSSION

### Molecular classification of APEC and NPEC isolates

Colibacillosis is a major infectious disease of poultry caused by APEC and is characterized by localized and systemic infections [1, 30, 41, 42]. The definition and classification of APEC remain subjects of debate; however, a multi-criteria approach incorporating the site of isolation and VAG profiles is increasingly accepted [1, 43]. Isolation of *E. coli* from extraintestinal organs, particularly the spleen, reflects the ability of the bacterium to invade host tissues, evade host defenses, and disseminate systemically. Detection in the spleen, an important peripheral lymphoid organ, is therefore considered a relevant biological indicator of APEC pathogenicity [33]. In addition, APEC strains typically harbor multiple VAGs and PAIs that contribute to their pathogenic potential [10, 13, 15, 16, 44, 45]. The mPCR assay has consequently become a widely used rapid and reliable molecular approach for detecting VAGs and characterizing APEC strains [46]. In the present study, 19 VAGs were investigated by mPCR: *ompT*, *iutA*, *fimC*, *iucD*, *hlyF*, *iss*, *iroN*, *irp2*, *fyuA*, *vat*, *papC*, *cva/cvi*, *astA*, *iucA*, *colV*, *tsh*, *eaeA*, *sfa_foc*, and *hlyA*.

Challenge experiments in 1-day-old chicks demonstrated that APEC strains harboring fewer than two of the five marker genes *hlyF*, *ompT*, *iroN*, *iss*, and *iutA* rarely exhibited pathogenicity, whereas most virulent APEC isolates carried two or more of these genes [40]. Furthermore, in a previous survey evaluating *ompT*, *hlyF*, *iroN*, *tsh*, *vat*, *iss*, *cvi/cva*, and *iucD* in chicken-derived *E. coli*, isolates carrying at least five of the investigated VAGs were classified as APEC, whereas those carrying fewer than five were considered NPEC [23]. Based on the classification criteria applied in the present study, 121/200 (60.5%) isolates were classified as APEC, whereas the remaining 79/200 (39.5%) were classified as NPEC.

Among the 121 APEC isolates, 115 (95.0%) originated from non-healthy chickens, whereas only 6/121 (5.0%) were recovered from clinically healthy birds. This distribution supports an association between the accumulation of VAGs and clinical colibacillosis. Nevertheless, recovery of APEC-classified isolates from healthy chickens indicates that clinically normal birds may carry *E. coli* strains with substantial virulence-associated repertoires. The presence of multiple VAGs alone may therefore be insufficient to determine whether clinical disease develops, because host susceptibility and environmental factors can also influence pathogenesis [1, 30, 43]. Clinically healthy chickens may consequently represent reservoirs of potentially pathogenic APEC within poultry populations [1, 30, 43].

### Diversity of VAGs

All 19 investigated VAGs were detected among *E. coli* isolates from non-healthy chickens, whereas 15 were detected among isolates from healthy chickens; *papC*, *eaeA*, *sfa_foc*, and *hlyA* were not detected in the latter group. Similarly, all 19 VAGs were represented among APEC isolates, whereas 14 were detected among NPEC isolates, with *papC*, *eaeA*, *sfa_foc*, *fyuA*, and *hlyA* absent from NPEC. These findings indicate that APEC isolates possessed a broader VAG repertoire than NPEC isolates [10, 23]. The accumulation of multiple VAGs appears to be an important feature of APEC pathogenic potential, consistent with previous studies reporting that highly virulent APEC strains generally harbor larger and more diverse virulence repertoires [3, 9, 10, 47]. This genetic complexity supports the value of VAG profiling as a molecular approach for differentiating APEC from less virulent or commensal *E. coli* populations in poultry [10, 20].

The exclusive detection of *papC*, *eaeA*, *sfa_foc*, *fyuA*, and *hlyA* among APEC isolates further suggests that these genes may contribute to the broader virulence repertoire observed in this group [2, 43]. Of particular One Health relevance, *papC*, *eaeA*, and *fyuA* have also been reported among human ExPEC, including uropathogenic *E. coli *(UPEC) and neonatal meningitis-associated *E. coli *(NMEC), where they are involved in adhesion, iron acquisition, and extraintestinal colonization [1, 30]. Their detection in poultry-derived APEC indicates that chickens may harbor *E. coli* strains possessing virulence determinants shared with human ExPEC. Although zoonotic transmission was not investigated in the present study, these findings support the importance of integrated surveillance across the animal–human–environment interface.

### Prevalence of major APEC-associated virulence genes

Among APEC isolates, the most frequently detected VAGs were *hlyF* (100.0%), *fimC* (96.7%), *iutA* (96.7%), *ompT* (95.9%), *iucD* (95.9%), *iss* (90.1%), *irp2* (79.3%), and *iroN* (65.3%) (Table 3). Previous studies have reported considerable variation in the prevalence of these genes, including *hlyF* (59.3%–100%), *fimC* (70.0%–94.9%), *ompT* (67.2%–100%), *iutA* (79.5%–93.1%), *iucD* (70.8%–100%), *iss* (55.6%–100%), *iroN* (67.88%–100%), and *irp2* (45.26%–100%) [9, 10, 14, 16, 18–20, 38, 44, 48–51]. In the present study, the prevalence of *ompT*, *iutA*, *hlyF*, *iss*, *iroN*, and *irp2* was generally comparable to previously reported ranges, whereas *fimC* occurred at a higher frequency.

The prevalence of the major APEC-associated VAGs in this study was generally comparable to or higher than that reported in other Asian countries [19, 22, 27, 52–56]. In particular, *fimC* (96.7%), *iutA* (96.7%), and *hlyF* (100.0%) were highly prevalent among APEC isolates and exceeded several previously reported estimates from China, South Korea, and Thailand. Similarly, *ompT* and *iss* occurred at frequencies within the upper ranges reported in Asian studies. In contrast, the prevalence of *iroN* (65.3%) was lower than that reported in some studies from China and South Korea but remained comparable to findings from India and Thailand (Supplementary Table S4).

The five-marker predictor set proposed by Johnson *et al.* [9], comprising *hlyF*, *ompT*, *iroN*, *iss*, and *iutA*, also showed substantial discriminatory value in the present study. Although individual markers occurred in some NPEC isolates, simultaneous carriage of all five markers was detected only among APEC isolates. These findings are consistent with those of Johnson *et al.* [9] and support the usefulness of this predictor set for molecular characterization and epidemiological surveillance of APEC in poultry [1, 43].

Previous studies from Vietnam reported lower frequencies of several individual VAGs, including *ompT* (28.7%), *iss* (22.5%), *hlyF* (18.7%), *iucD* (17.5%), and *iroN* (12.5%), than those observed in the present study [27]. Differences among studies may be related to sampling strategy, isolate source, study population, and diagnostic or classification criteria. For example, isolates obtained from fecal samples or healthy chickens may harbor fewer VAGs than isolates recovered from systemic infection sites such as the liver or spleen [2, 43].

### Distribution of less prevalent VAGs

The remaining VAGs among APEC isolates occurred at frequencies ranging from 4.1% to 57.0% (Table 3). The prevalence of *fyuA* (57.0%) and *tsh* (30.6%) was lower than that reported in China (77.8% and 61.8%, respectively) [10]. In contrast, *vat* (48.8%) and *papC* (38.0%) occurred more frequently than previously reported in China (27.01% and 18.98%, respectively) [19] but less frequently than in Australia (89.7% and 44.8%, respectively) [48].

The frequencies of *cva/cvi* (40.5%), *iucA* (34.7%), and *colV* (31.4%) were lower than several previously reported estimates, whereas the prevalence of *astA* (36.4%) fell within the broad range reported in earlier studies [10, 48–50]. Conversely, *eaeA* (21.5%), *sfa_foc* (15.7%), and *hlyA* (4.1%) were detected more frequently than in several previous reports, in which prevalence estimates of approximately 1.28%, 0%–4.4%, and 0%–1.28%, respectively, were reported [10, 48–50]. The prevalence of *fyuA*, *tsh*, *papC*, *vat*, *cva/cvi*, and *iucA* has varied considerably among studies, whereas *astA* and *hlyA* have generally occurred at relatively low frequencies in reports from China, South Korea, India, Thailand, and Vietnam (Supplementary Table S4).

Notably, *eaeA* and *sfa_foc*, which have rarely been reported among APEC isolates in Asian studies, were detected in a subset of the Vietnamese isolates [10, 19, 22, 27, 44, 52–56]. Their occurrence contributes to the broader VAG diversity observed in the study population, although molecular detection alone cannot establish their direct contribution to pathogenicity. Differences in VAG prevalence among APEC populations from different countries may reflect geographical variation, poultry production systems, host populations, chicken breeds, circulating bacterial lineages, sampling strategies, and differences in the VAG panels investigated [30, 48]. Such genetic variability may contribute to differences in pathogenic potential among APEC populations [57] and is epidemiologically relevant for understanding disease severity and strain circulation within poultry production systems.

### Virulence marker combinations and pathogenic potential

APEC strains are commonly characterized by multiple marker genes associated with adhesion, iron acquisition, serum resistance, proteolysis, and other virulence-related functions. These include *ompT* (episomal outer membrane protease), *iutA* (ferric aerobactin receptor), *fimC* (Type 1 fimbrial adhesin), *iucD* (aerobactin synthesis), *hlyF* (putative avian hemolysin), *iss* (increased serum survival), and *iroN* (salmochelin siderophore receptor) [9, 14]. APEC isolates recovered from chickens with clinical signs and lesions of colibacillosis frequently harbor multiple combinations of these genes [9, 37, 44]. Ewers *et al.* [14] identified eight VAGs, *papC*, *iucD*, *irp2*, *tsh*, *vat*, *astA*, *iss*, and *cva/cvi*, as useful markers for differentiating APEC from NPEC. In the present study, APEC isolates simultaneously harbored between one and eight of these markers.

Previous studies have also demonstrated that the number and combination of VAGs are associated with differences in pathogenic potential. Highly pathogenic *E. coli* strains have been reported to harbor approximately 8–13 VAGs, including *irp2*, *fyuA*, *iutA*, *papC*, *iss*, *colV*, *iucD*, *iucA*, *fimA*, *fimC*, *hlyA*, *tsh*, and *colBM*, whereas strains with intermediate or low-pathogenicity generally harbor fewer virulence determinants [10]. Jeong *et al.* [18] reported *iroN* as the most prevalent gene among Korean APEC isolates (100%), followed by *fimC* (94.1%), *ompT* (90.1%), *hlyF* (87.1%), *iss* (78.2%), *iucD* (73.3%), *tsh* (61.4%), *fyuA* (44.6%), *irp2* (43.6%), and *vat* (10.9%). These isolates were classified into 27 molecular pathotypes, with *iroN-fimC-ompT-hlyF-iucD-tsh-iss-irp2-fyuA*, *iroN-fimC-ompT-hlyF-iucD-tsh-iss*, and *iroN-fimC-ompT-hlyF-iss* predominating.

In the present study, 103/121 (85.1%) APEC isolates harbored more than eight VAGs and were distributed among 18 combination patterns (Tables 4 and 5; Figure 5), whereas the remaining 18/121 isolates (14.9%) contained 5–7 VAGs and were distributed among five patterns. Necropsy examination of diseased chickens revealed characteristic lesions of colibacillosis, including perihepatitis, pericarditis, airsacculitis, swollen-head syndrome, salpingitis, and osteomyelitis/synovitis, and APEC isolates recovered from spleen samples showed substantial diversity in their VAG profiles [58]. Notably, 23/121 APEC isolates (19.0%) harbored 14–15 VAGs and were recovered from chickens exhibiting typical and severe colibacillosis-associated lesions [1]. Of these, eight isolates (6.6%) simultaneously carried 15 VAGs, whereas 15 isolates (12.4%) carried 14 VAGs.

These extensive virulence repertoires are consistent with the concept that APEC pathogenic potential reflects the combined action of multiple virulence determinants rather than the presence of a single gene. Similar multi-gene combinations have been reported predominantly among highly virulent APEC strains [2, 10, 18, 23, 46]. The coexistence of genes involved in adhesion, iron acquisition, toxin production, serum resistance, and plasmid-associated functions may collectively facilitate colonization, survival in extraintestinal tissues, evasion of host defenses, and systemic dissemination [1, 3, 26, 30]. Comparable VAG-rich profiles have previously been associated with severe colibacillosis and increased pathogenicity in experimental models [1, 3, 10, 30]. Nevertheless, the present study did not experimentally determine virulence; therefore, isolates with extensive VAG repertoires should be regarded as having putatively greater pathogenic potential. Confirmation would require *in vivo* pathogenicity testing, such as chicken challenge experiments or embryo lethality assays [9, 10].

### Comparison with previous studies in Vietnam

The present findings are consistent with previous reports from Vietnam. Vounba *et al.* [27] detected several APEC/ExPEC-associated VAGs, including *iroN*, *iss*, *hlyF*, *ompT*, and *iucD*, among *E. coli* isolates from healthy chickens, together with β-lactam resistance determinants, demonstrating the coexistence of virulence and antimicrobial resistance traits in poultry-associated *E. coli*. Similarly, Sary *et al.* [28] identified multidrug-resistant ExPEC-like *E. coli* harboring VAGs such as *papC* and *tsh* in retail chicken carcasses, indicating the potential for dissemination through the poultry food chain. More recently, Hoang *et al.* [29] reported that the Vietnamese APEC strain O78:H9 (HPVN24) possessed a broad repertoire of virulence and antimicrobial resistance genes.

Consistent with these studies, the APEC isolates investigated here showed a high prevalence of core VAGs, including *iroN*, *iss*, *ompT*, and *hlyF*. However, unlike previous Vietnamese investigations that primarily focused on antimicrobial resistance, selected virulence determinants, or individual strains, the present study characterized 19 VAGs across a larger isolate collection and identified 23 distinct VAG combination patterns, including profiles containing up to 15 genes. These findings extend current knowledge of the molecular diversity of poultry-associated APEC in Vietnam and provide a broader basis for epidemiological surveillance.

### Co-occurrence of VAGs

The co-occurrence heatmap revealed strong associations among several VAGs in APEC isolates, including *hlyF*, *ompT*, *iss*, *iroN*, *iutA*, *cva/cvi*, and *colV*. These genes include determinants associated with iron acquisition, serum resistance, bacterial adaptation, and systemic dissemination and are frequently linked to ColV-associated virulence plasmids [1, 3, 30]. In contrast, NPEC isolates showed a less extensive and more fragmented co-occurrence network, consistent with their more limited VAG repertoires. The UpSet plot similarly demonstrated that VAGs occurred in more numerous and complex combinations among APEC than among NPEC isolates.

The frequent co-occurrence of these determinants supports the importance of considering combinations of VAGs rather than individual genes when characterizing APEC. However, because plasmid analysis was not performed, the present data cannot confirm that the observed gene associations were physically located on the same ColV-like plasmids. Further plasmid characterization and whole-genome sequencing would be required to establish their genomic organization and patterns of co-inheritance.

### One Health implications

Several VAGs detected in the present study, including *papC*, *fyuA*, *iutA*, *iroN*, *ompT*, and *iss*, overlap with virulence signatures reported in human ExPEC lineages, particularly UPEC and NMEC. Previous studies have demonstrated that APEC and human ExPEC may share virulence determinants, plasmid-associated pathogenicity genes, and, in some cases, phylogenetic backgrounds such as ST95 and ST131 [1, 30]. The detection of complex multi-gene VAG profiles among Vietnamese poultry APEC isolates, including strains carrying 12–15 VAGs, therefore indicates the presence of isolates with ExPEC-associated molecular characteristics.

These findings have potential One Health relevance, particularly in poultry-producing regions where humans, poultry, poultry products, and farm environments are closely interconnected. However, this study did not directly investigate zoonotic transmission, and detecting shared VAGs alone does not establish transmission between poultry and humans. Genomic characterization and comparative phylogenetic analyses of poultry, human, and environmental isolates would be required to determine whether epidemiologically related lineages circulate across these compartments [1, 30, 59, 60]. Nevertheless, the findings support continued molecular surveillance of APEC virulence profiles together with antimicrobial resistance monitoring within an integrated One Health framework.

### Limitations and future research directions

Several limitations should be considered when interpreting these findings. First, pathogenic potential was inferred from VAG profiles and clinical or pathological associations because *in vivo* challenge experiments were not performed. Consequently, the presence of multiple VAGs should not be interpreted as definitive evidence of virulence. Second, serotyping, phylogrouping, multilocus sequence typing (MLST), whole-genome sequencing, and plasmid characterization were not undertaken, limiting assessment of clonal relationships, genomic backgrounds, and the physical organization of co-occurring VAGs. Third, exclusive use of spleen samples may have introduced sampling bias by preferentially representing systemic infections rather than the broader diversity of *E. coli* colonizing poultry. Finally, the cross-sectional design did not permit evaluation of temporal changes, persistence, or transmission dynamics.

Despite these limitations, this study provides baseline information on the prevalence, diversity, combination profiles, and co-occurrence patterns of APEC-associated VAGs in northern Vietnam. Future studies should integrate experimental pathogenicity assessment with serotyping, phylogrouping, MLST, whole-genome sequencing, plasmid analysis, and antimicrobial resistance profiling. Comparative genomic studies incorporating poultry, human, food, and environmental *E. coli* isolates would also help clarify transmission pathways and the One Health relevance of the VAG-rich APEC lineages identified in this study.

## CONCLUSION

This study demonstrated substantial diversity and complexity in VAG profiles among poultry-associated *E. coli* isolates from northern Vietnam. Of 515 spleen samples collected from chickens across 35 farms, 200 (38.8%) yielded *E. coli*, including 160/365 (43.8%) samples from non-healthy chickens and 40/150 (26.7%) samples from healthy chickens. Based on the molecular classification criteria applied in this study, 121/200 (60.5%) isolates were classified as APEC and 79/200 (39.5%) as NPEC. APEC was detected on 34/35 (97.1%) surveyed farms, and 115/121 (95.0%) APEC isolates originated from non-healthy chickens. All 19 investigated VAGs were represented among APEC isolates, with *hlyF*, *fimC*, *iutA*, *ompT*, *iucD*, and *iss* being particularly prevalent. APEC isolates also exhibited greater diversity and complexity of VAG combinations than NPEC isolates, with 23 distinct profiles identified. Notably, 23/121 (19.0%) APEC isolates harbored 14–15 VAGs, including eight isolates carrying 15 VAGs. The five-marker predictor set *hlyF*, *ompT*, *iroN*, *iss*, and *iutA* was simultaneously detected in 70/121 (57.8%) APEC isolates but in none of the NPEC isolates.

The marked differences in VAG prevalence and combination patterns between APEC and NPEC isolates support the application of multi-gene molecular profiling for epidemiological characterization of poultry-associated *E. coli*. The high occurrence of APEC among non-healthy chickens and its detection on nearly all surveyed farms emphasize the importance of strengthening farm-level surveillance and colibacillosis prevention programs in northern Vietnam. Detection of APEC-classified isolates in clinically healthy chickens further indicates that apparently healthy birds may harbor potentially pathogenic strains. Moreover, the presence of ExPEC-associated determinants, including *papC*, *fyuA*, *iutA*, *iroN*, *ompT*, and *iss*, supports integrated surveillance of poultry, humans, food products, and farm environments within a One Health framework, although this study did not directly assess zoonotic transmission.

A major strength of this study was the characterization of 19 VAGs in 200 *E. coli* isolates obtained from 515 chickens across 35 farms in five major poultry-producing provinces, together with comparisons between APEC and NPEC isolates and analyses of VAG combinations and co-occurrence patterns.

The findings demonstrate a broad and complex repertoire of VAGs among APEC circulating in northern Vietnam and distinguish these isolates molecularly from NPEC populations. The predominance of VAG-rich profiles among APEC, together with their widespread occurrence in the surveyed poultry farms, highlights the epidemiological importance of these strains in poultry production. These baseline data provide a foundation for improved molecular surveillance and future genomic and pathogenicity studies aimed at strengthening colibacillosis control and assessing the broader One Health significance of poultry-associated APEC in Vietnam.

## DATA AVAILABILITY

The datasets generated and/or analyzed during the current study are included in the article and its supplementary files. Supplementary files and raw data supporting the findings of this study are available from the corresponding author upon reasonable request.

## GENERATIVE AI DECLARATION

During the preparation of this manuscript, ChatGPT (OpenAI, GPT-5.5) was used solely to improve the English language, grammar, readability, and overall presentation of the manuscript. All AI-assisted content was critically reviewed, edited, and verified by the authors. The authors take full responsibility for the accuracy, originality, interpretation, and scientific integrity of the manuscript. No generative AI tool was used for data generation, statistical analysis, interpretation of results, or scientific decision-making.

## AUTHORS’ CONTRIBUTIONS

QLT and VTL: Contributed equally to this work and share first authorship. QLT and VTL: Conceptualization, study design, statistical analysis, and writing–original draft preparation. HAD, QLT, TMLH, and VTL: Designed the data collection tools. VTL, HAD, THGT, NVT, and TTL: Field sampling, data collection, and laboratory investigation. HAD, QLT, TMLH, THGT, TNV, and TTL: Data interpretation, writing–review and editing, and critical revision of the manuscript for important intellectual content. All authors have read, contributed to, and approved the final manuscript, and agree to be accountable for all aspects of the work.

## References

[1] Kathayat D, Lokesh D, Ranjit S, Rajashekara G (2021). Avian pathogenic *Escherichia coli* (APEC): an overview of virulence and pathogenesis factors, zoonotic potential, and control strategies. Pathogens.

[2] Khairullah AR, Afnani DA, Riwu KHP, Widodo A, Yanestria SM, Moses IB (2024). Avian pathogenic *Escherichia coli*: epidemiology, virulence and pathogenesis, diagnosis, pathophysiology, transmission, vaccination, and control. Vet World.

[3] Ovi F, Zhang L, Nabors H, Jia L, Adhikari P (2023). A compilation of virulence-associated genes that are frequently reported in avian pathogenic *Escherichia coli* (APEC) compared to other *E. coli*. J Appl Microbiol..

[4] Lu Q, Zhang W, Luo L, Wang H, Shao H, Zhang T (2022). Genetic diversity and multidrug resistance of phylogenic groups B2 and D in InPEC and ExPEC isolated from chickens in Central China. BMC Microbiol.

[5] Ewers C, Janssen T, Wieler LH (2003). [Avian pathogenic *Escherichia coli* (APEC)]. Berl Munch Tierarztl Wochenschr.

[6] Denamur E, Clermont O, Bonacorsi S, Gordon D (2021). The population genetics of pathogenic *Escherichia coli*. Nat Rev Microbiol.

[7] Barros MR, Silveira WD, Costa MM (2025). Prevalence of virulence genes in *Escherichia coli* isolates from poultry farms in Northeastern Brazil. Cienc Rural..

[8] McMullin PF (2020). Diseases of poultry 14th edition. Avian Pathol.

[9] Johnson TJ, Wannemuehler Y, Doetkott C, Johnson SJ, Rosenberger SC, Nolan LK (2008). Identification of minimal predictors of avian pathogenic *Escherichia coli* virulence for use as a rapid diagnostic tool. J Clin Microbiol.

[10] Wang J, Tang P, Tan D, Wang L, Zhang S, Qiu Y (2015). The pathogenicity of chicken pathogenic *Escherichia coli* is associated with the numbers and combination patterns of virulence-associated genes. Open J Vet Med.

[11] Vandekerchove D, Vandemaele F, Adriaensen C, Zaleska M, Hernalsteens JP, De Baets L (2005). Virulence-associated traits in avian *Escherichia coli*: comparison between isolates from colibacillosis-affected and clinically healthy layer flocks. Vet Microbiol.

[12] Janben T, Schwarz C, Preikschat P, Voss M, Philipp HC, Wieler LH (2001). Virulence-associated genes in avian pathogenic *Escherichia coli* (APEC) isolated from internal organs of poultry having died from colibacillosis. Int J Med Microbiol.

[13] Kaper JB, Nataro JP, Mobley HL (2004). Pathogenic *Escherichia coli*. Nat Rev Microbiol.

[14] Ewers C, Janssen T, Kiessling S, Philipp HC, Wieler LH (2005). Rapid detection of virulence-associated genes in avian pathogenic *Escherichia coli* by multiplex polymerase chain reaction. Avian Dis.

[15] Rodriguez-Siek KE, Giddings CW, Doetkott C, Johnson TJ, Fakhr MK, Nolan LK (2005). Comparison of *Escherichia coli* isolates implicated in human urinary tract infection and avian colibacillosis. Microbiology (Reading).

[16] Rodriguez-Siek KE, Giddings CW, Doetkott C, Johnson TJ, Nolan LK (2005). Characterizing the APEC pathotype. Vet Res.

[17] Dziva F, Stevens MP (2008). Colibacillosis in poultry: unravelling the molecular basis of virulence of avian pathogenic *Escherichia coli* in their natural hosts. Avian Pathol.

[18] Jeong YW, Kim TE, Kim JH, Kwon HJ (2012). Pathotyping avian pathogenic *Escherichia coli* strains in Korea. J Vet Sci.

[19] Afayibo DJA, Zhu H, Zhang B, Yao L, Abdelgawad HA, Tian M (2022). Isolation, molecular characterization, and antibiotic resistance of avian pathogenic *Escherichia coli* in Eastern China. Vet Sci..

[20] Subedi M, Luitel H, Devkota B, Bhattarai RK, Phuyal S, Panthi P (2018). Antibiotic resistance pattern and virulence genes content in avian pathogenic *Escherichia coli* (APEC) from broiler chickens in Chitwan, Nepal. BMC Vet Res.

[21] Fujimoto Y, Inoue H, Kanda T, Ijiri M, Uemura R (2021). Virulence-associated gene profiles of *Escherichia coli* isolated from chickens with colibacillosis in Japan and their correlation with pathogenicity in chicken embryos. Avian Dis.

[22] Young MM, de Oliveira AL, Nolan LK, Barbieri NL, Logue CM (2022). Identification of novel genes involved in the biofilm formation process of avian pathogenic *Escherichia coli* (APEC). PLoS One.

[23] Johar A, Al-Thani N, Al-Hadidi SH, Dlissi E, Mahmoud MH, Eltai NO (2021). Antibiotic resistance and virulence gene patterns associated with avian pathogenic *Escherichia coli* (APEC) from broiler chickens in Qatar. Antibiotics (Basel). 2021;10(5).

[24] De Carli S, Ikuta N, Lehmann FK, da Silveira VP, de Melo Predebon G, Fonseca AS (2015). Virulence gene content in *Escherichia coli* isolates from poultry flocks with clinical signs of colibacillosis in Brazil. Poult Sci.

[25] Hub GOHP (2023). Poultry in Vietnam. Available from: onehealthpoultry.org. 2023.

[26] Nawaz S, Wang Z, Zhang Y, Jia Y, Jiang W, Chen Z (2024). Avian pathogenic *Escherichia coli* (APEC): current insights and future challenges. Poult Sci.

[27] Vounba P, Arsenault J, Bada-Alambédji R, Fairbrother JM (2019 Apr). Pathogenic potential and the role of clones and plasmids in beta-lactamase-producing *E. coli* from chicken faeces in Vietnam. BMC Vet Res..

[28] Sary K, Fairbrother JM, Arsenault J, de Lagarde M, Boulianne M (2019). Antimicrobial resistance and virulence gene profiles among *Escherichia coli* isolates from retail chicken carcasses in Vietnam. Foodborne Pathog Dis.

[29] Hoang MD, Lanh PT, Hien VT, Kao CY, Quyen DV Comprehensive genomic and phenotypic characterization of *Escherichia coli* O78:H9 strain HPVN24 isolated from diarrheic poultry in Vietnam. Microorganisms. 2025;13(10).

[30] Hu J, Afayibo DJA, Zhang B, Zhu H, Yao L, Guo W (2022). Characteristics, pathogenic mechanism, zoonotic potential, drug resistance, and prevention of avian pathogenic *Escherichia coli* (APEC). Front Microbiol.

[31] Naing L, Nordin RB, Abdul Rahman H, Naing YT (2022). Sample size calculation for prevalence studies using Scalex and ScalaR calculators. BMC Med Res Methodol.

[32] Awawdeh L, Forrest R, Turni C, Cobbold R, Henning J, Gibson J (2022). Risk factors associated with the carriage of pathogenic *Escherichia coli* in healthy commercial meat chickens in Queensland, Australia. Poultry.

[33] Nakamura K, Maeda M, Imada Y, Imada T, Sato K (1985). Pathology of spontaneous colibacillosis in a broiler flock. Vet Pathol.

[34] Bej AK, DiCesare JL, Haff L, Atlas RM (1991). Detection of *Escherichia coli* and *Shigella* spp. in water by using the polymerase chain reaction and gene probes for uid. Appl Environ Microbiol.

[35] Shehata AMAA, Hershan AA (2016). Boiling, freezing-thawing and simple chemical extraction methods are comparable to costly kit-based bacterial DNA extraction methods as evidenced by conventional and real-time PCR. Egypt J Med Microbiol.

[36] Ahmed OB, Dablool AS (2017). Quality improvement of the DNA extracted by boiling method in Gram negative bacteria. Int J Bioassays.

[37] Paton AW, Paton JC (1998). Detection and characterization of Shiga toxigenic *Escherichia coli* by using multiplex PCR assays for *stx1, stx2, eaeA,* enterohemorrhagic *E. coli hlyA, rfbO111,* and *rfbO157*. J Clin Microbiol.

[38] Ewers C, Janssen T, Kiessling S, Philipp HC, Wieler LH (2004). Molecular epidemiology of avian pathogenic *Escherichia coli* (APEC) isolated from colisepticemia in poultry. Vet Microbiol.

[39] de Oliveira AL, Rocha DA, Finkler F, de Moraes LB, Barbieri NL, Pavanelo DB (2015). Prevalence of ColV plasmid-linked genes and in vivo pathogenicity of avian strains of *Escherichia coli*. Foodborne Pathog Dis.

[40] Le Bouguenec C, Archambaud M, Labigne A (1992). Rapid and specific detection of the pap, afa, and sfa adhesin-encoding operons in uropathogenic *Escherichia coli* strains by polymerase chain reaction. J Clin Microbiol.

[41] Ha EJ, Hong SM, Choi KS, Kwon HJ (2025). Evolution and zoonotic risk of O1:K1 and O2:K1 avian pathogenic *Escherichia coli*. Microbes Infect.

[42] Jhandai P, Mittal D, Gupta R, Kumar M (2025). An insight into newly emerging avian pathogenic *E. coli* serogroups, biofilm formation, ESBLs and integron detection and in vivo pathogenicity in chicken. Microb Pathog.

[43] Joseph J, Zhang L, Adhikari P, Evans JD, Ramachandran R (2023). Avian pathogenic *Escherichia coli* (APEC) in broiler breeders: an overview. Pathogens.

[44] Kim YB, Yoon MY, Ha JS, Seo KW, Noh EB, Son SH (2020). Molecular characterization of avian pathogenic *Escherichia coli* from broiler chickens with colibacillosis. Poult Sci.

[45] Knöbl T, Moreno AM, Paixão R, Gomes TA, Vieira MA, da Silva Leite D (2012). Prevalence of avian pathogenic *Escherichia coli* (APEC) clone harboring sfa gene in Brazil. ScientificWorldJournal.

[46] Suttitas T (2024). Extended-spectrum beta-lactamase (ESBL) production and virulence genes profile of avian pathogenic *Escherichia coli* (APEC) isolated from broiler chickens in eastern Thailand. Vet Integr Sci.

[47] Mehat JW, van Vliet AHM, La Ragione RM (2021). The avian pathogenic *Escherichia coli* (APEC) pathotype is comprised of multiple distinct, independent genotypes. Avian Pathol.

[48] Awawdeh L, Forrest R, Turni C, Cobbold R, Henning J, Gibson J (2024). Virulence-associated genes in faecal and clinical *Escherichia coli* isolates cultured from broiler chickens in Australia. Aust Vet J.

[49] Jamshidi A, Razmyar J, Fallah N (2016). Detection of eaeA, hlyA, stx1 and stx2 genes in pathogenic *Escherichia* coli isolated from broilers affected with colibacillosis. Iran J Vet Med.

[50] Johnson TJ, Wannemuehler Y, Johnson SJ, Stell AL, Doetkott C, Johnson JR (2008). Comparison of extraintestinal pathogenic *Escherichia coli* strains from human and avian sources reveals a mixed subset representing potential zoonotic pathogens. Appl Environ Microbiol.

[51] Johnson TJ, Siek KE, Johnson SJ, Nolan LK (2006). DNA sequence of a ColV plasmid and prevalence of selected plasmid-encoded virulence genes among avian *Escherichia coli* strains. J Bacteriol.

[52] Thomrongsuwannakij T, Blackall PJ, Djordjevic SP, Cummins ML, Chansiripornchai N (2020). A comparison of virulence genes, antimicrobial resistance profiles and genetic diversity of avian pathogenic *Escherichia coli* (APEC) isolates from broilers and broiler breeders in Thailand and Australia. Avian Pathol.

[53] Laopiem S, Witoonsatian K, Kulprasetsri S, Panomwan P, Pathomchai-Umporn C, Kamtae R (2025). Antimicrobial resistance, virulence gene profiles, and phylogenetic groups of *Escherichia coli* isolated from healthy broilers and broilers with colibacillosis in Thailand. BMC Vet Res.

[54] Narasinakuppe Krishnegowda D, Singh BR, Mariappan AK, Munuswamy P, Singh KP, Monalisa S (2022). Molecular epidemiological studies on avian pathogenic *Escherichia coli* associated with septicemia in chickens in India. Microb Pathog.

[55] Patel SS, Patel AC, Mohapatra SK, Chauhan HC, Sharma KK, Shrimali MD (2024). Antibiotic resistance and virulence gene patterns associated with multi drug resistant avian pathogenic *Escherichia coli* (APEC) isolated from broiler chickens in India. Indian J Microbiol.

[56] Yongyod R, Eiamsam-Ang T, Kamolrat N, Srisawat S, Walanan H, Chaisaeng S (2026). Fluoroquinolone-resistant avian pathogenic *Escherichia coli* isolated from asymptomatic broiler chickens in a slaughterhouse in Northern Thailand. Pathogens.

[57] Hasan RN, Jasim SA, Ali YH Detection of fimH, kpsMTII, hlyA, and traT genes in *Escherichia coli* isolated from Iraqi patients with cystitis. Gene Reports. 2022.

[58] Kazimierczak J, Pospiech K, Sowińska P, Pękala A, Borówka P, Wójcik EA (2025). A rapid detection of avian pathogenic *Escherichia coli* (APEC) strains based on minimal number of virulence markers identified by whole genome sequencing. BMC Microbiol.

[59] Palmieri N, Apostolakos I, Paudel S, Hess M (2023). The genetic network underlying the evolution of pathogenicity in avian *Escherichia coli*. Front Vet Sci.

[60] Watts A, Wigley P (2024). Avian pathogenic *Escherichia coli*: an overview of infection biology, antimicrobial resistance and vaccination. Antibiotics (Basel).

